# Inequality on the frontline: A multi-country study on gender differences in mental health among healthcare workers during the COVID-19 pandemic

**DOI:** 10.1017/gmh.2024.18

**Published:** 2024-03-04

**Authors:** Diana Czepiel, Clare McCormack, Andréa T.C. da Silva, Dominika Seblova, Maria F. Moro, Alexandra Restrepo-Henao, Adriana M. Martínez, Oyeyemi Afolabi, Lubna Alnasser, Rubén Alvarado, Hiroki Asaoka, Olatunde Ayinde, Arin Balalian, Dinarte Ballester, Josleen A.l. Barathie, Armando Basagoitia, Djordje Basic, María S. Burrone, Mauro G. Carta, Sol Durand-Arias, Mehmet Eskin, Eduardo Fernández-Jiménez, Marcela I. F. Frey, Oye Gureje, Anna Isahakyan, Rodrigo Jaldo, Elie G. Karam, Dorra Khattech, Jutta Lindert, Gonzalo Martínez-Alés, Franco Mascayano, Roberto Mediavilla, Javier A. Narvaez Gonzalez, Aimee Nasser-Karam, Daisuke Nishi, Olusegun Olaopa, Uta Ouali, Victor Puac-Polanco, Dorian E. Ramírez, Jorge Ramírez, Eliut Rivera-Segarra, Bart P.F. Rutten, Julian Santaella-Tenorio, Jaime C. Sapag, Jana Šeblová, María T. S. Soto, Maria Tavares-Cavalcanti, Linda Valeri, Marit Sijbrandij, Ezra S. Susser, Hans W. Hoek, Els van der Ven

**Affiliations:** 1 Parnassia Psychiatric Institute, Parnassia Groep, The Hague, The Netherlands; 2 Clinical, Neuro- and Developmental Psychology, Amsterdam Public Health Institute, Vrije Universiteit Amsterdam, Amsterdam, The Netherlands; 3 Department of Child and Adolescent Psychiatry, New York University Langone Medical Center, New York, NY, USA; 4 Faculty of Medicine, University of São Paulo, São Paulo, Brazil; 5 Faculty of Medicine Santa Marcelina, São Paulo, Brazil; 6 Second Faculty of Medicine, Charles University, Prague, Czech Republic; 7 Department of Epidemiology, Columbia University Mailman School of Public Health, New York, NY, USA; 8 Epidemiology Group, National School of Public Health, University of Antioquia, Medellín, Colombia; 9 Department of Psychiatry, University College Hospital, Ibadan, Nigeria; 10 Population Health Research Section, King Abdullah International Medical Research Center (KAIMRC), King Saud Bin Abdulaziz University for Health Sciences (KSAU-HS), Riyadh, Saudi Arabia; 11 Department of Public Health, School of Medicine, University of Valparaíso, Valparaiso, Chile; 12 School of Public Health, University of Chile, Santiago, Chile; 13 Department of Psychiatric Nursing, Graduate School of Medicine, The University of Tokyo, Tokyo, Japan; 14 Department of Psychiatry, University of Ibadan, Ibadan, Nigeria; 15 Question Driven Design and Analysis Group, New York, NY, USA; 16 University Hospital, Federal University of Rio Grande, Rio Grande, Brazil; 17 Institute for Development, Research, Advocacy and Applied Care (IDRAAC), Beirut, Lebanon; 18 Unidad de Investigación, Consultora Salud Global Bolivia, Sucre, Bolivia; 19 Institute of Health Sciences, Universidad de O’Higgins, Rancagua, Chile; 20 Department of Medical Sciences and Public Health, University of Cagliari, Cagliari, Italy; 21 National Institute of Psychiatry Ramon de la Fuente Muñiz, Mexico City, Mexico; 22 Department of Psychology, Koç University, Istanbul, Turkey; 23 Department of Psychiatry, Clinical Psychology and Mental Health, La Paz University Hospital, Madrid, Spain; 24 Hospital La Paz Institute for Health Research (IdiPAZ), Madrid, Spain; 25 European University of Madrid, Madrid, Spain; 26 Social and Community Academic Unit, University of Chubut, Chubut, Argentina; 27 National Institute of Health Named After Academician S. Avdalbekyan, Yerevan, Armenia; 28 Department of Psychiatry and Clinical Psychology, Faculty of Medicine, University of Balamand, Beirut, Lebanon; 29 Department of Psychiatry and Clinical Psychology, St George Hospital University Medical Center, Beirut, Lebanon; 30 Department Psychiatry A, Razi Hospital La Manouba, Manouba, Tunisia; 31 Faculty of Health and Social Work, University of Applied Sciences Emden/Leer, Emden, Germany; 32 Department of Epidemiology, Harvard T.H. Chan School of Public Health, Boston, MA, USA; 33 New York State Psychiatric Institute, New York, NY, USA; 34 Department of Psychiatry, Universidad Autónoma de Madrid, Madrid, Spain; 35 Centro de Investigación Biomédica en Red de Salud Mental (CIBERSAM), Carlos III Health Institute, Madrid, Spain; 36 Instituto de Investigación Sanitaria del Hospital Universitario La Princesa, Madrid, Spain; 37 Maestría en Epidemiología, División de Postgrados, El Bosque University, Bogotá, Colombia; 38 Departamento de Salud Pública, Facultad de Medicina, Universidad Nacional de Colombia, Sede Bogotá, Bogotá, Colombia; 39 Department of Mental Health, Graduate School of Medicine, The University of Tokyo, Tokyo, Japan; 40 Department of Oral and Maxillofacial Surgery, University College Hospital, Ibadan, Nigeria; 41 Faculty of Medicine of Tunis, University of Tunis El Manar, Tunis, Tunisia; 42 Departments of Health Policy & Management and Epidemiology & Biostatistics, Downstate Health Sciences University, Brooklyn, NY, USA; 43 Faculty of Medical Sciences, University of San Carlos of Guatemala, Guatemala City, Guatemala; 44 School of Behavioral and Brain Sciences, Ponce Research Institute, Ponce Health Sciences University, Ponce, Puerto Rico; 45 Department of Psychiatry and Neuropsychology, School for Mental Health and Neuroscience, Maastricht University Medical Centre, Maastricht, The Netherlands; 46 Department of Clinical Epidemiology and Biostatistics, Pontifical Xavierian University, Bogotá, Colombia; 47 Department of Public Health and Family Medicine, Pontificia Universidad Católica de Chile, Santiago, Chile; 48 Dalla Lana School of Public Health, University of Toronto, Toronto, ON, Canada; 49 Centre for Addiction and Mental Health, Toronto, ON, Canada; 50 Emergency Department, Motol University Hospital, Prague, Czech Republic; 51 Czech Society for Emergency and Disaster Medicine, Czech Medical Association of J. E. Purkyně, Prague, Czech Republic; 52 Dirección de Investigación Ciencia y Tecnología, Universidad San Francisco Xavier de Chuquisaca, Sucre, Bolivia; 53 School of Medicine and Psychiatric Institute, Federal University of Rio de Janeiro, Rio de Janeiro, Brazil; 54 Department of Biostatistics, Mailman School of Public Health, Columbia University, New York, NY, USA; 55 WHO Collaborating Centre for Research and Dissemination of Psychological Interventions, Amsterdam, The Netherlands; 56 University Medical Center Groningen, University Center of Psychiatry, University of Groningen, Groningen, The Netherlands

**Keywords:** COVID-19, healthcare workers, gender differences, healthcare disparities, mental health, cross-country

## Abstract

Healthcare workers (HCWs) were at increased risk for mental health problems during the COVID-19 pandemic, with prior data suggesting women may be particularly vulnerable. Our global mental health study aimed to examine factors associated with gender differences in psychological distress and depressive symptoms among HCWs during COVID-19. Across 22 countries in South America, Europe, Asia and Africa, 32,410 HCWs participated in the COVID-19 HEalth caRe wOrkErS (HEROES) study between March 2020 and February 2021. They completed the General Health Questionnaire-12, the Patient Health Questionnaire-9 and questions about pandemic-relevant exposures. Consistently across countries, women reported elevated mental health problems compared to men. Women also reported increased COVID-19-relevant stressors, including insufficient personal protective equipment and less support from colleagues, while men reported increased contact with COVID-19 patients. At the country level, HCWs in countries with higher gender inequality reported less mental health problems. Higher COVID-19 mortality rates were associated with increased psychological distress merely among women. Our findings suggest that among HCWs, women may have been disproportionately exposed to COVID-19-relevant stressors at the individual and country level. This highlights the importance of considering gender in emergency response efforts to safeguard women’s well-being and ensure healthcare system preparedness during future public health crises.

## Impact statement

In the wake of the COVID-19 pandemic, our research sheds light on the global impact of the pandemic on the mental health of high-risk populations, such as healthcare workers and especially women. Contextual and ecological factors, such as gender inequality and COVID-19 mortality rates in each country, emerged as potential contributors to mental health outcomes. Our findings reveal that women worldwide were disproportionally exposed to various stressors related to COVID-19 both at the individual and country level. We have identified several factors contributing to the less favorable mental health outcomes observed among women, namely less access to sufficient personal protective equipment, less perceived support from colleagues and higher country COVID-19 mortality rates. This study underscores the need for gender-informed policies and interventions to develop effective and equitable emergency preparedness and relief programs. By acknowledging and addressing the mental health challenges women may face, we can provide better support for their specific needs. This, in turn, might help mitigate the exacerbation of gender inequalities in mental health and enhance the resilience of healthcare systems in the face of global health crises.

## Introduction

Healthcare workers (HCWs) in both clinical and non-clinical roles have been at the frontlines of the battle against COVID-19. Workplace conditions contribute to physician burnout, anxiety and depression symptoms even outside a pandemic context (Brooks et al., 2011). Healthcare work during a pandemic, in combination with disruptions to daily life, may further contribute to an environment that results in especially high levels of psychosocial stress. Indeed, multiple studies have demonstrated that HCWs have been experiencing high levels of depression, anxiety and psychological distress throughout the COVID-19 pandemic (Sahebi et al., 2021; Aymerich et al., 2022; Chen et al., 2022). Mental health problems not only have implications for HCWs’ well-being but also challenge the effectiveness of healthcare systems by negatively affecting the workforce, making this not only an occupational hazard but a public health threat recognized by the World Health Organization (WHO, 2021).

Women have been consistently found to experience greater rates of mental health problems in most populations (Luo et al., 2020; Penninx et al., 2022). Research on mental health consequences following disasters has demonstrated that women experience higher rates of subsequent posttraumatic stress disorder, anxiety and depression than men (Galea et al., 2005; Bell and Folkerth, 2016). These pre-existing gender differences in mental health problems were exacerbated following the onset of the COVID-19 pandemic (Borrescio-Higa and Valenzuela, 2021; Martínez Pajuelo et al., 2022; Penninx et al., 2022; Sun et al., 2023), with women reporting a greater increase in mental health problems than men (Witteveen et al., 2023). Gender imbalance in healthcare and other workforce sectors (Gupta et al., 2019; Hay et al., 2019), combined with longstanding inequalities in unpaid domestic work, potentially contributed to the gendered effects of the economic crisis and lockdown restrictions during the initial waves of the COVID-19 pandemic (OECD, 2020; The Lancet, 2020; Borrescio-Higa and Valenzuela, 2021; Mele et al., 2021). The “mental load” that disproportionately burdens women, particularly those with children, is increasingly being recognized as a form of unpaid cognitive and emotional labor that presents an additional burden beyond physical domestic labor and has especially received public attention since the COVID-19 pandemic (Craig and Churchill, 2021; Dean et al., 2022). Structural workplace factors, such as the significant underrepresentation of women in managerial and senior positions in healthcare (Boniol et al., 2019; OECD, 2020), are also implicated in the disproportionate hardship experienced by women during the pandemic. Hence, developing effective strategies to protect HCWs from adverse mental health outcomes requires an evaluation of mental health problems differentially by gender in addition to the assessment of the overall impact of COVID-19 on HCWs’ mental health.

Contextual country-level characteristics may affect the degree to which HCWs are exposed to pandemic-relevant stressors, since the scale of the outbreak and the nature of the public health response has varied dramatically between countries (Luo et al., 2020; Vizheh et al., 2020; Bollyky et al., 2022). HCWs from low, lower-middle and upper-middle-income countries, in particular, are not well represented in research. Moreover, the widespread global nature of the COVID-19 pandemic presents a unique opportunity to examine the role of structural, modifiable factors in shaping the mental health of the HCW workforce. Outcomes of research examining such factors will provide valuable data with clear implications for policy.

To our knowledge, no other study has examined gender differences in the relation between COVID-19-relevant exposures and HCWs’ mental health at a global scale. We seek to address these gaps in order to inform tailored policy and interventions for those affected by the COVID-19 pandemic and to guide preparedness efforts for future emergencies involving HCWs (Chandan et al., 2020). Improved organizational and national responses to crises can support gender-equitable policies, subsequently improving health outcomes for people of all genders (Heymann et al., 2019). This is of particular significance for women, considering that their needs have not been sufficiently incorporated into emergency response or preparedness (Davies and Bennett, 2016).

The main aim of this study was to examine the extent to which individual and country-level factors are associated with gender differences in psychological distress and depressive symptoms among HCWs during the early stages of the COVID-19 pandemic, and to explore the consistency of these differences across countries. We investigated three domains of COVID-19-relevant exposures which might be related to gender differences in mental health, namely work-related, interpersonal and country-level factors. In addition, we examined whether there were different patterns in gender differences in the relationship between COVID-19-relevant exposures and mental health across countries.

## Methods

### Study design and participants

The international study titled “COVID-19 HEalth caRe wOrkErS (HEROES)” (Mascayano et al., 2022) sought to assess the impact of the COVID-19 pandemic on the mental health of HCWs. The target population included HCWs with both clinical and non-clinical roles (e.g., nurses, physicians, psychologists, dentists, managers, administrative staff, security, cleaning staff) who were employed across a broad spectrum of healthcare facilities (e.g., hospitals, primary care centers, mental health facilities, elderly homes, rehabilitation centers, emergency medical service). To be eligible for inclusion, participants needed to be of legal age and employed in either public or private healthcare settings. The present study employed a cross-sectional research design and the sample (*N* = 32,410) was recruited between March 2020 and February 2021, with different recruitment dates across countries. In the majority of countries, recruitment took place during the initial wave of the pandemic, at a time when vaccines were scarcely available and information about the disease transmission was unclear (Mascayano et al., 2022). A map of the participating countries can be found in Supplementary Figure S1.

In most countries, healthcare centers were recruited based on convenience sampling; however, in São Paulo (Brazil), Colombia, Lebanon, Japan and Belgium facilities were randomly selected. Sites invited participants to join by providing a link to the study’s digital platform through their work email address or the internal communication system of the healthcare center. The study used a secure platform, following the Research Electronic Data Capture (REDCap) model (Harris et al., 2009, 2019), that was designed to ensure data security and quality. This system was created *ad hoc* to facilitate translation into different languages, and thus the survey was available in all the languages of the participants (English, Spanish, Arabic, Italian, Dutch, Japanese, Armenian, German, Portuguese and Czech). More details about the recruitment method have been previously described elsewhere (Mascayano et al., 2022).

### Measures

Sociodemographic characteristics included age, gender, participants’ and their parents’ highest level of completed education, and presence of mental health problems or a chronic physical illness before the pandemic (see Supplementary Table S1).

#### Depressive symptoms

For the assessment of depressive symptoms, we used the 9-item Patient Health Questionnaire (PHQ-9), a self-report instrument commonly utilized as a depression screening tool (Spitzer et al., 2000). It has been validated in several languages and has been widely used in most of the countries of the present study. The questionnaire comprises items that correspond to the symptoms of major depressive disorder, rated on a four-point Likert scale (0–3). In this study, internal consistency (α =.89) was found to be good (Taber, 2018). We used the inter-country validated cut-off ≥ 10 as an indication of the possible presence of a depressive disorder (Kroenke et al., 2001).

#### Psychological distress

We used the 12-item General Health Questionnaire (GHQ-12), a screening tool validated in many languages and commonly used in most of the countries of the current study to assess general psychological distress (Goldberg et al., 1997). This self-report instrument is unidimensional and comprises items designed to evaluate the presence of symptoms of psychological distress experienced during the past week, rated on a four-point Likert scale (0–3). Participant scores were determined using the Likert scoring method (0–1–2–3). We observed good internal consistency (Taber, 2018) in this study (α =. 85). We used the cut-off ≥ 15 to classify participants as presenting with psychological distress (Lundin et al., 2017).

#### Individual-level (work-related and interpersonal) exposures

Items created *ad hoc* were used to measure COVID-19-relevant exposures, that is, being in contact with COVID-19 patients, considering personal protective equipment (PPE) to be sufficient, interpersonal adversity (experience of discrimination, interpersonal conflict or violence) and perceived support from colleagues. Creating and translating these items was a collaborative effort among native-speaking researchers in different regions. The items with their response categories can be found in Supplementary Table S1.

#### Country-level exposures

To measure regional COVID-19 severity, we calculated average COVID-19 mortality rates during each country’s recruitment period (mortality rates = deaths/country population × 100,000; average mortality rates = mortality rates/number of recruitment days in each country) based on the confirmed COVID-19 deaths as reported by Johns Hopkins University’s COVID-19 Data Repository (Dong et al., 2020). The Gender Inequality Index (GII) published by the World Health Organization (WHO) and the United Nations Development Programme (UNDP, 2022) was used to capture differences in gender inequality across countries. The GII indicates inequality in achievements between women and men across five dimensions (maternal mortality ratio, adolescent birth rate, share of seats in parliament, population with at least some secondary education and labor force participation rate). GII ranges from 0 to 1, with higher values signifying greater inequality. Each country’s total GII as published for 2021 (UNDP, 2022) was utilized, except for Puerto Rico, where GII was unavailable because of its relationship with the United States (an unincorporated territory of the United States). In sensitivity analyses we included the World Bank’s income classification for the fiscal year 2020. A country’s income is defined based on the gross national income per capita, calculated using the World Bank Atlas method, which results in one of the following classifications: low income, lower-middle income, upper-middle income and high income (Fantom and Serajuddin, 2016). Venezuela’s classification was derived from the fiscal year 2019, as data for 2020 was unavailable.

### Statistical analyses

Complete and non-complete cases were compared in terms of sociodemographic and clinical correlates and mental health outcomes. To compare COVID-19-relevant exposures among men and women, we conducted Wilcoxon rank-sum tests for continuous and Pearson’s chi-square tests for categorical variables. We used cut-off scores to dichotomize the presence of depressive symptoms and psychological distress. Following the Sex and Gender Equity in Research (SAGER) guidelines (Heidari et al., 2016), we reported descriptive statistics for participants identifying as “other gender” (rather than men or women), but we did not perform any further analyses or any interpretations, due to the low sample size (*n* = 58). Multiple imputations were performed in R (version 4.2.0) using Chained Random Forests (MissRanger package) to deal with missing data. The number of trees was set to 128, based on recommendations by Oshiro and colleagues (Oshiro et al., 2012); the model converged in 3 iterations. All other analyses were conducted using Stata (version 17.0); an alpha level of. 05 was used for all statistical tests.

Choropleth maps and forest plots were generated to illustrate differences in mental health outcomes between men and women across countries. To this end, Poisson regressions were performed to obtain incidence rate ratios and 95% confidence intervals. The meta-analysis command in STATA software was used to calculate the random-effects models. The website https://app.datawrapper.de was used to create the maps. Logistic regression models were performed to assess the association between work-related (contact with COVID-19 patients, considering PPE to be sufficient), interpersonal (interpersonal adversity, support from colleagues) and country-level predictors (COVID-19 mortality rates, gender inequality) and mental health outcomes (depressive symptoms and psychological distress). Associations are reported as unadjusted and adjusted (i.e., correcting for confounding variables and the remaining work-related, interpersonal and country-level predictors) with odds ratios (ORs) and 95% confidence intervals (CI). We performed three sets of sensitivity analyses. First, we repeated logistic regression analyses accounting for country income. Second, models were conducted separately for physicians and nurses to account for the confounding factor of occupation. Other occupation categories were highly heterogeneous, limiting comparability. Third, we used multilevel logistic regression models to explore differences in mental health outcomes by gender, and their relationship with exposures at the individual and country level (see Supplementary Material).

## Results

Gender differences in sociodemographic characteristics and COVID-19-relevant exposures are presented in Table 1. Women in our sample were more likely than men to have parents with a lower completed education level, be employed as “other HCWs” (i.e., respiratory or physical therapist, first responder, dietician), nurses or health technicians, and to report having previous mental health problems. Men were more likely than women to be employed as physicians or ancillary HCWs (i.e., secretary, cleaning or maintenance staff) and were of slightly older age on average. There were no gender differences in reported physical illness. Regarding COVID-19-relevant exposures, women were more likely than men to report receiving insufficient support from colleagues, experiencing at least one form of interpersonal adversity, considering PPE to be insufficient, and being inhabitants of a country with higher gender inequality, higher COVID-19 mortality rates and upper-middle income. Men, on the other hand, were more likely than women to report having contact with COVID-19 patients, experiencing all three measured forms of interpersonal adversity, and living in a country with high or low-middle income. Women and men were equally likely to complete the survey; other differences between complete and non-complete cases can be found in Supplementary Table S2.

**Table 1.** Sociodemographic characteristics, individual and country-level exposure variables by gender

	Women (*n* = 23,167)	Men (*n* = 8,140)	*χ* ^2^/ Ws
Sociodemographic characteristics			
Age, Mdn (IQR)	38 (31–47)^a^	39 (31–50)^b^	68110584.50, *p* <. 001
Mother’s education, *n* (%)			87.53, *p* <. 001
(Incomplete) primary school	7,123 (30.74)^a^	2,255 (27.71)^b^	
Secondary school	5,432 (23.45)^a^	1,819 (22.35)^a^	
Technical-professional training	4,140 (17.87)^a^	1,209 (14.85)^b^	
Undergraduate degree	3,366 (14.53)^a^	1,214 (14.91)^b^	
Postgraduate studies	2,120 (9.15)^a^	925 (11.36)^b^	
Does not apply	633 (2.73)^a^	205 (2.52)^a^	
Missing	353 (1.52)	513 (6.30)	
Father’s education, *n* (%)			151.57, *p* <. 001
(Incomplete) primary school	6,513 (28.12)^a^	1,971 (24.21)^b^	
Secondary school	4,910 (21.19)^a^	1,523 (18.71)^b^	
Technical-professional training	4,169 (18.00)^a^	1,266 (15.55)^b^	
Undergraduate degree	3,462 (14.94)^a^	1,261 (15.49)^b^	
Postgraduate studies	2,766 (11.94)^a^	1,303 (16.01)^b^	
Does not apply	973 (4.20)^a^	298 (3.66)^a^	
Missing	374 (1.61)	518 (6.36)	
Occupation, *n* (%)			1.03, *p* <. 001
Physicians	5,539 (23.91)^a^	3,254 (39.98)^b^	
Nurses	5,503 (23.75)^a^	1,008 (12.38)^b^	
Health technicians	2,308 (9.96)^a^	582 (7.15)^b^	
Ancillary HCWs	2,163 (9.34)^a^	865 (10.63)^b^	
Other HCWs	6,613 (28.54)^a^	1,997 (24.53)^b^	
Missing	1,041 (4.49)	434 (5.33)	
Chronic physical illness, *n* (%)			2.82, *p* =. 244
No	12,274 (52.98)^a^	4,160 (51.11)^a^	
Yes	3,203 (13.83)^a^	1,065 (13.08)^a^	
Missing	7,690 (33.20)	2,915 (35.82)	
Previous mental health problems, *n* (%)			20.43, *p* <. 001
No	13,835 (59.72)^a^	4,804 (59.02)^b^	
Yes	1,325 (5.72)^a^	347 (4.26)^b^	
Missing	8,007 (34.56)	2,989 (36.72)	
Individual level			
Contact with COVID-19 patients, *n* (%)			17.88, *p* <. 001
No	7,250 (31.29)^a^	2,448 (30.07)^b^	
Yes	10,524 (45.43)^a^	3,882 (47.69)^b^	
I do not know	3,821 (16.49)^a^	1,218 (14.96)^b^	
Missing	1,572 (6.79)	592 (7.27)	
Personal protective equipment, *n* (%)^c^			74.12, *p* <. 001
Insufficient	10,958 (47.30)^a^	3,538 (43.46)^b^	
Sufficient	9,575 (41.33)^a^	3,742 (45.97)^b^	
Not applicable	658 (2.84)^a^	148 (1.82)^b^	
Missing	1,976 (8.53)	712 (8.75)	
Number of types of experienced interpersonal adversity, *n* (%)^d^			63.20, *p* <. 001
0	9,427 (40.69)^a^	3,624 (44.52)^b^	
1	6,677 (28.82)^a^	2,136 (26.24)^b^	
2	3,448 (14.88)^a^	1,036 (12.73)^b^	
3	1,349 (5.82)^a^	548 (6.73)^b^	
Missing	2,266 (9.78)	796 (9.78)	
Support from colleagues, *n* (%)^e^			11.52, *p =.* 001
Low	3,687 (15.91)^a^	1,717 (14.39)^b^	
High	14,553 (62.82)^a^	5,244 (64.42)^b^	
Missing	4,927 (21.27)	1,725 (21.19)	
Country level			
Gender inequality, *n* (%)			47.03, *p* <. 001
Lowest tertile	8,161 (35.5)^a^	3,202 (39.7)^b^	
Middle tertile	8,633 (37.6)^a^	2,902 (35.9)^b^	
Highest tertile	6,180 (26.9)^a^	1,966 (24.4)^b^	
COVID-19 mortality rate, *n* (%)			227.94, *p* <. 001
Lowest tertile	7,323 (31.6)^a^	3,321 (40.8)^b^	
Middle tertile	7,896 (34.1)^a^	2,355 (28.9)^a^	
Highest tertile	7,948 (34.3)^a^	2,464 (30.3)^b^	
Country income, *n* (%)^f^			96.47, *p* <. 001
Lower-middle income	848 (3.7)^a^	366 (4.5)^b^	
Upper-middle income	12,052 (52)^a^	3,725 (45.8)^b^	
High-income	10,267 (44.3)^a^	4,049 (49.7)^b^	

*Note: χ* ^2^ has been calculated based on valid entries only. Ancillary HCWs: e.g., non-clinical manager, administrator/secretary/admission, patient transportation, food/hospitality, cleaning staff, maintenance staff, security staff, student, statistician, analyst, IT, health information management. Other HCWs: e.g., clinical manager, psychologist, social worker, physical therapist, respiratory therapist, speech therapist, occupational therapist, first responder, midwife, dentist, dentist assistant, dietician, doctor assistant, epidemiologist/public health, pharmacist, community worker, primary attention worker, health promotion/prevention, health educator.

a,b Values not sharing the same subscript are significantly different from each other.

c This continuous variable was dichotomized for descriptive purposes as follows: “no, they are completely insufficient,” “no, they are very insufficient” and “no, they are somewhat insufficient” = “insufficient,” “yes, they are sufficient” = “sufficient.”

d This is a composite variable consisting of three separate variables: experienced stigma or discrimination, experienced problems with family members of COVID-19 patients, experienced violence. The variables were first dichotomized (experienced/not experienced) and then merged to indicate the number of types of interpersonal adversity HCWs have experienced.

e This ordinal variable was dichotomized for descriptive purposes as follows: “strongly disagree” and “disagree” = “low,” “agree” and “strongly agree” = “high.”

f In the case of Venezuela, the classification for the fiscal year 2019 was used due to the unavailability of a classification for the year 2020. Furthermore, the low-income category was omitted due to the absence of participating countries falling within the low-income bracket in our study.

Among HCWs in the current study, 21% (23% women, 17% men) reported depressive symptoms and 41% (47% women, 40% men) experienced psychological distress. Sample sizes, age, gender and occupation distribution per country and for the entire sample are given in Supplementary Table S3. The GII, average COVID-19 mortality rates and classification by income per country are presented in Supplementary Table S4.

**Figure 1.**
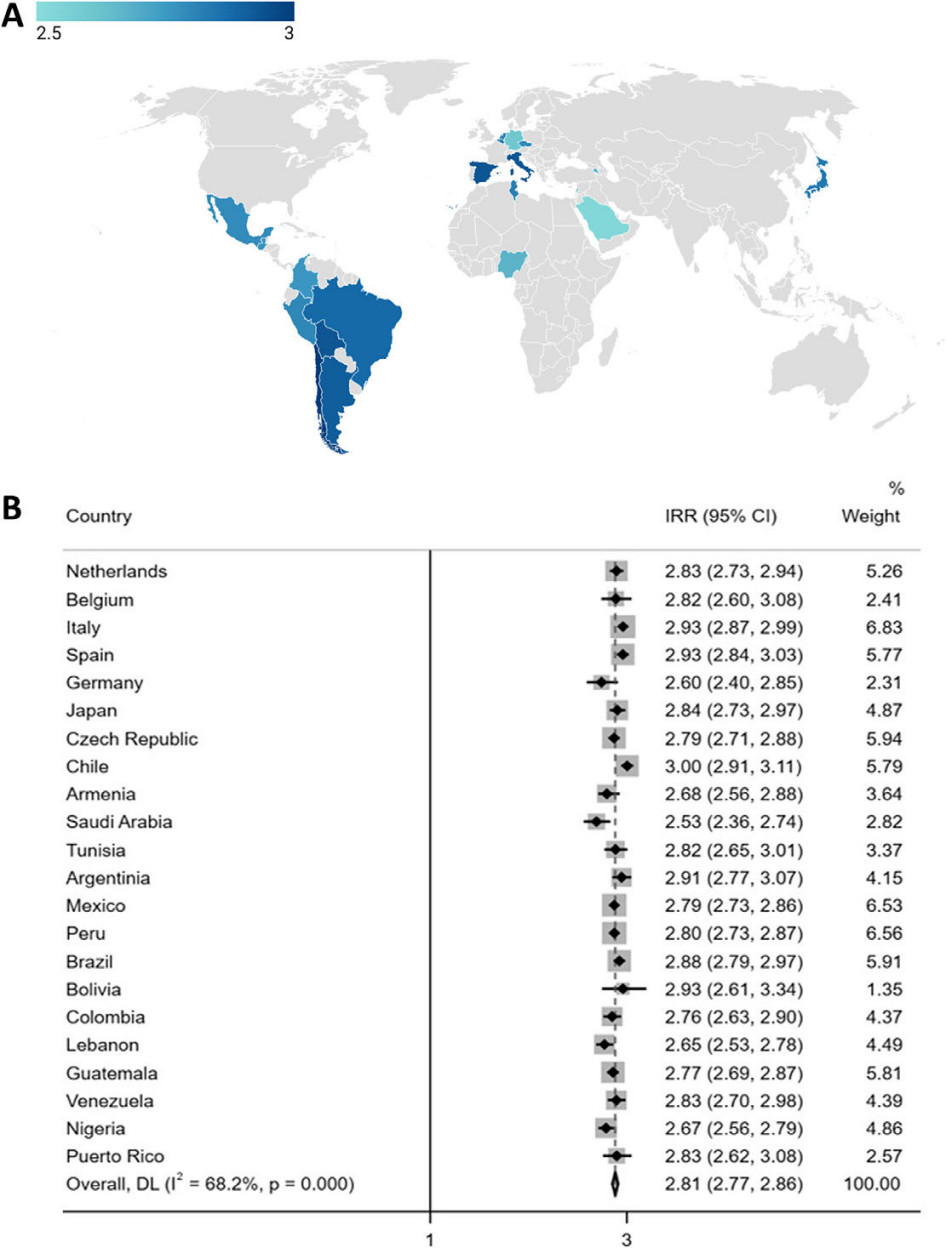
Incidence rate ratio (IRR) of depressive symptoms for women compared to men across countries (A); the intensity of the color corresponds to the IRR, with darker shades indicating higher IRR values. (B) IRRs listed according to the Gender Inequality Index of countries in ascending order. IRR’s range from 2.5 to 3.

Choropleth maps and forest plots were created to explore gender differences in depressive symptoms and psychological distress across countries (see Supplementary Material). Consistently elevated depressive symptoms and psychological distress were found among women compared to men, yet there was heterogeneity between countries (see Figure 1 and Supplementary Figure S2).

Unadjusted and adjusted ORs (uOR and aOR, respectively) along with the 95% CIs for the associations between COVID-19-relevant exposures and depressive symptoms among women and men are listed in Table 2. Women were more likely to report elevated depressive symptoms than men. Both women and men were more likely to report depressive symptoms when they had been in contact with COVID-19 patients, if they considered the provided PPE to be insufficient, had experienced discrimination, interpersonal conflict or violence and did not consider their colleagues to be supportive. Both men and women living in countries with higher gender inequality were less likely to report depressive symptoms. COVID-19 mortality rates were not associated with depressive symptoms among women or men.

**Table 2.** Odds ratios and 95% confidence intervals [OR (95% CI)] for depressive symptoms for the entire sample and stratified by gender

	Entire sample			
	Unadjusted OR (95% CI)		Adjusted OR (95% CI)	
Gender (reference category = men)	1.52** (1.42–1.62)		1.48** (1.37–1.60)	

	Women		Men	
	Unadjusted OR (95% CI)	Adjusted OR (95% CI)	Unadjusted OR (95% CI)	Adjusted OR(95% CI)
Work-related factors				
Contact with COVID-19 patients	1.66** (1.54–1.78)	1.52** (1.40–1.66)	1.85** (1.60–2.13)	1.37** (1.16–1.62)
Insufficient PPE	1.14** (1.11–1.18)	1.10** (1.06–1.14)	1.24** (1.17–1.31)	1.10* (1.02–1.18)
Interpersonal factors				
Interpersonal adversity	1.55** (1.50–1.60)	1.49** (1.43–1.55)	1.78** (1.68–1.88)	1.54** (1.44–1.66)
Unsupportive colleagues	1.48** (1.42–1.53)	1.43** (1.37–1.50)	1.66** (1.55–1.78)	1.55** (1.43–1.69)
Country-level factors				
Gender inequality	.69** (.57–.84)	.14** (.11–19)	1.58*** (1.10–2.21)	.30** (.19–.49)
COVID-19 mortality rates	1.37** (1.17–1.62)	.95 (.77–1.18)	1.87** (1.39–2.53)	.86 (.58–1.26)

*Note:* Adjusted OR = Odds ratio adjusted for age, mother’s education, father’s education, occupation, chronic physical illness and previous mental health problems and for all other predictors in the table.

*p <. 05; **p <. 001.

When analyses were repeated for psychological distress (Supplementary Table S5), the same pattern of findings was observed, with one exception in the adjusted models: women living in countries with higher mortality rates were more likely to report psychological distress.

In sensitivity analyses accounting for country income (see Supplementary Table S6), the effect estimates were similar, except for two comparisons: (a) men living in countries with a higher GII were no longer more likely to report depressive symptoms (while women still were) and (b) not only women, as observed in the primary analyses, but also men living in countries with higher COVID-19 morality rates were more likely to report psychological distress.

Regression models performed separately for physicians and nurses showed that among nurses, multiple gender differences that were observed in the full sample were attenuated and were no longer statistically significant, while associations remained largely unchanged for physicians. The findings are displayed in Supplementary Table S7 and elaborated upon in the Supplementary Material.

Results of multilevel models examining gender differences in mental health outcomes and their relation to exposures at the individual and country levels confirmed the findings of the regression analyses. Women had a higher likelihood of reporting depressive symptoms and psychological distress than men. Despite consistent associations between predictors and mental health outcomes with regression models at the country level, no significant main effects were found. However, a statistically significant interaction indicated that women in countries with higher gender inequality reported fewer depressive symptoms and psychological distress compared to men (see Supplementary Table S8 and description in the Supplementary Material).

## Discussion

In this large multi-country sample of 32,410 HCWs, we examined gender differences in self-reported mental health outcomes and COVID-19-relevant exposures at the individual and country levels. We identified significant gender differences in the severity of exposure to all the examined stressors, with women reporting increased exposure to most of the stressors, including less access to sufficient PPE, increased exposure to stigma, discrimination or violence and less perceived support from colleagues compared to men. This may have contributed to the elevated psychological distress and depressive symptoms among women compared to men across all countries, which may generalize to explain worsened mental health among women overall. All work-related and interpersonal exposures were associated with poorer mental health both among men and women. At the country level, lower levels of mental health problems were observed among HCWs working in countries characterized by higher levels of gender inequality, while increased psychological distress was observed among women residing in countries with higher COVID-19 mortality rates.

### Gender differences in COVID-19-relevant exposures and relation to mental health

We found that women were less likely to perceive PPE as sufficient, in line with previous warnings that PPE originally designed for men may not adequately protect women, or that women may disproportionately face structural barriers to accessing this equipment (McMahon et al., 2008; Rose and Rae, 2019). As anticipated, HCWs’ perceptions of having sufficient PPE were found to have a protective association with depressive symptoms and psychological distress, corroborating the existing literature that suggests that HCWs are more likely to report mental health problems when they perceive the provided PPE to be insufficient (Khajuria et al., 2021; Moitra et al., 2021; Sharma et al., 2021). Many countries, in particular those with a lower income, faced PPE shortages especially at the beginning of the pandemic (Burki, 2020). Ensuring PPE availability is an occupational health imperative to prevent infections among HCWs; here we additionally demonstrate that insufficient PPE represents a psychological stressor contributing to elevated psychological distress and depressive symptoms in the context of a pandemic. An unexpected finding related to virus exposure was that men more often reported having direct contact with COVID-19 patients than women. This was counter to expectations, since nurses are often the staff in closest contact with patients and are more likely to be women. This finding may be explained by the relatively high percentage of men in our sample employed as physicians with direct patient contact, and the fact that the majority of women were “other HCWs” (e.g., speech therapist, midwife, health educator), which did not necessarily involve contact with COVID-19 patients.

An unfortunate reality for many HCWs – particularly in the initial wave of the COVID-19 pandemic when uncertainty and fear were high – was exposure to stigma, discrimination and even violence from the public due to their status as frontline workers at the center of the crisis (Billings et al., 2021). Disparities in exposure to these stressors may also contribute to observed gender differences in mental health outcomes: in our sample of HCWs, women were more likely to report experiencing interpersonal adversity, a finding that coincides with literature highlighting that stigma reflects and reinforces existing social inequalities (Brewis et al., 2020). This may reflect the relatively vulnerable social status of women that persists globally, and that women are more likely to be victims of violence and interpersonal conflict in general. Perceived discrimination, interpersonal conflict or violence among HCWs in the total sample were related to both increased depressive symptoms and psychological distress, adding to a growing body of literature that links stigmatization and discrimination during the COVID-19 pandemic to mental health problems among HCWs (Zolnikov and Furio, 2020; Moro et al., 2022; Saragih et al., 2022). During the COVID-19 pandemic negative impacts on the mental health of women may have, therefore, been compounded by the long-standing public health crisis of gender-based violence, which in this case was expressed as interpersonal conflict and discrimination due to their status as frontline workers.

Lockdown measures, social distancing and quarantining because of the COVID-19 pandemic had a profound social impact (Antiporta and Bruni, 2020). Redeployment to a new team or department was also frequent, resulting in HCWs being separated from their familiar social network and peers (Billings et al., 2021). Among HCWs, women seem to have been disproportionately affected, as they reported experiencing less support from their colleagues compared to men. This was a surprising finding, as women generally report higher and more meaningful perceived support compared to men (Turner and Brown, 2010; Kneavel, 2021). It should be noted that perceived social support typically presents a stronger association with mental health than actually received social support (Turner and Brown, 2010), suggesting that cognitive appraisal plays a significant role, which can also be influenced by depressive symptoms. Social support is a strategy often used by women to cope with stress (Meléndez et al., 2012) and being restricted in – or even deprived of – the possibility to make use of this coping strategy due to the social restrictions and/or high workload could decrease its demonstrated protective role in ameliorating stress.

### Relation of COVID-19-relevant exposures at the country level to mental health

While gender differences in mental health outcomes were present across all countries, we noted high heterogeneity of this effect across countries. Accordingly, we leveraged our multi-country sample to examine whether key factors at country level contributed to observed gender differences in mental health outcomes. First, in line with our hypothesis, higher COVID-19 mortality rates were associated with increased psychological distress among women but not among men. There was no significant association between country mortality rates and depressive symptoms. Considering our sample consists entirely of HCWs, high mortality may represent a particularly potent stressor as not only are individuals more likely to face loss and grief in their community, but also face increased exposure to patient death in the workplace – a major contributor to mental health problems (Mosheva et al., 2021). Women may have experienced a stronger emotional response to facing patients die than men, consistent with findings from an earlier study in the COVID-19 pandemic showing more pronounced associations between witnessing patient death and symptoms of posttraumatic stress among women (Mosheva et al., 2021). Another possibility is that emotional labor involved in healthcare work in sensitive scenarios, such as being exposed to grieving families, may disproportionately fall on women due to this labor being perceived as “women’s jobs” (Gray, 2010). Rather than interpreting the current finding as an inherent vulnerability of women, it is argued that power relations and social roles ascribed to women disproportionately affect their abilities to face crises (Smyth and Sweetman, 2015).

Another country-level exposure we examined was gender inequality, defined according to the WHO Gender Inequality Index (GII). Contrary to expectations and to existing literature (Yu, 2018; Pacheco et al., 2019), both men and women working in countries with higher gender inequality reported lower scores on psychological distress and depressive symptoms. A possible explanation is mental health stigma, which is known to be more prevalent in lower- and middle-income countries (Semrau et al., 2014). As a country’s economic growth and gender inequality are correlated (Cuberes and Teignier, 2014), it could be hypothesized that HCWs living in countries with higher gender inequality were more likely to underreport or conceal mental health problems due to experienced or anticipated stigma. Furthermore, our sample consists only of HCWs: a group with overall higher education and economic status than the general population, which could have acted as a buffer for the negative consequences of gender inequality at the country level. The current finding could also be indicative of a self-selection effect, as our sample is limited to HCWs and therefore not representative of the general population. It is possible that in countries with greater gender inequality, women working in the healthcare sector are more likely to be of higher socio-economic status or possess greater individual strengths and resilience to cope with selection pressures at the workplace, while more vulnerable individuals might be more likely to be self-selected out of the workforce.

While not all associations between COVID-19-relevant exposures at the country level and mental health outcomes were confirmed in different analyses, a consistent finding was that women in countries with higher gender inequality reported less mental health complaints. A possible explanation is offered by Hopcroft and Bradley (2007), who suggest that women living in more gender-unequal countries have low expectations of gender equality, which may lead them to not report, acknowledge or experience distress even if their actual position in the society is disadvantageous. This explanation coincides with literature about dysfunctional aspects of resilience, suggesting that in certain contexts individuals learn to adapt to and tolerate disparity and inequalities (Mahdiani and Ungar, 2021).

Finally, when analyses were repeated among nurses only, many of the observed associations were comparable to the entire sample but were no longer statistically significant. Notably, among nurses only, women no longer reported more mental health problems than men, and the relation between contact with COVID-19 patients and mental health became non-significant only among men. It has been previously found that, in comparison to other HCWs, nurses face a heightened risk of experiencing mental health issues during the COVID-19 pandemic (Zhang et al., 2022), with some studies finding an even more increased risk among women compared to men in this group (Simonetti et al., 2021; Varghese et al., 2021), which is not consistent with our findings. In our sample there was considerable overlap between gender and occupation roles since most nurses (84.4%) were women. The fact that differences were still found between nurses and the entire sample of HCWs potentially suggests a different response to pandemic-relevant exposures. It is possible that mental health among nurses is more closely related to aspects of their occupational role and working conditions during the pandemic than to gender. Alternatively, the different pattern of findings among the subgroup of nurses may also be driven by reduced statistical power due to the small number of men (15.5%). Future research with more equal gender representation across HCW occupation should try to disentangle the gendered effects on mental health.

### Strengths and limitations

HEROES included HCWs with both clinical and non-clinical roles, as well as low-, lower-middle-, upper-middle- and high-income countries, which addresses a key gap in research on the mental health of HCWs who are often underrepresented in research. We adopted a gender-based approach to look beyond the seemingly poorer mental health among women and attempted to explain these findings by considering disproportionate exposure to stress factors. Moreover, we examined contextual, country-level exposures, such as gender inequality and COVID-19 severity. We also attempted to separate the effect of gender and HCW occupation, which is often intertwined, by performing separate analyses for physicians and nurses.

Findings must be considered in the context of several potential limitations. First, the current study was based on convenience sampling for most countries; additionally, only physicians and nurses were included in comparative analyses exploring occupation-based differences. Selection bias may have limited the generalizability of the findings to the broader group of HCWs. Additionally, no data prior to the pandemic was available from our sample, and the current analyses are cross-sectional, which renders it impossible to establish causal relations as gender differences pre-date the pandemic. Also, countries participated during different stages of the pandemic, as the recruitment period began in March 2020 and ended in February 2021. Representation of gender diversity was enhanced by inclusion of the category “other gender,” however the sample size identifying as belonging to this category was low and did not allow us to draw any conclusions about this underrepresented group. In terms of outcome measures, the PHQ-9 and the GHQ-12 were offered in the primary language of the respective country; however, the translated versions have not been validated in all countries, and optimal cut-off scores may differ per country, which might limit comparability. This may be reflected in some country-level inconsistencies. Finally, gender is known to intersect with other social categories, such as class, race, age, sexuality, wealth and religion, contributing to relative social advantages and disadvantages (Gupta et al., 2019); yet we did not take an intersectional approach to modeling these factors given constraints of our non-representative sample.

### Implications

In a complex crisis such as a pandemic, this convergence of social and environmental factors results in an exacerbation of pre-existing gender disparities in strain and mental health, possibly intensifying gender-related mental health challenges globally. We identified several key factors contributing to the poorer mental health outcomes observed among women: decreased availability of sufficient PPE; increased exposure to discrimination and interpersonal conflict; less perceived support from colleagues and a more pronounced mental health impact of country-level COVID-19 mortality. The unique mental health consequences that women may experience following the onset of a public health crisis, such as the COVID-19 pandemic, highlight the urgent need to develop emergency response policies and intervention strategies that take gender into account. Identifying the extent to which an emergency affects men and women differently is a prerequisite for creating effective and equitable programs for disaster preparedness and relief. Emergency response efforts that address the needs of women could potentially alleviate the deterioration of mental health resulting from the pandemic and mitigate the exacerbation of gender inequalities in mental health. Safeguarding the mental health of HCWs by adopting a gender perspective ensures that healthcare systems are better prepared for future waves of COVID-19 or other pandemics.

Addressing gender differences would require evidence-based, multicomponent approaches, including but not limited to (a) providing education to decision-makers, HCWs and the public on gender equality at the workplace and the community; (b) redesigning work environments and procedures; (c) promoting gender-sensitive policies and organizational culture; (d) guaranteeing access to mental health support and services to all HCWs, according to the differentiated needs of women and men; (e) investing resources in data collection, analysis and research on mental health needs by gender, as well as preparedness, prevention and response during crises and (f) raising awareness about gender differences in mental health among HCWs of all genders.

Future studies may include a more representative sample, use a longitudinal design ideally with a pre-pandemic or pre-crisis assessment, and structured interviews to ascertain the existence of clinically significant mental health problems. Moreover, future research should focus on HCWs with other gender identities, who are known to be an even greater risk factor for adverse mental health outcomes (Plöderl and Tremblay, 2015), as well as to examine the intersection of gender and social inequalities.

## Supporting information

Czepiel et al. supplementary material Czepiel et al. supplementary material

## Data Availability

For use of the data, proposals can be sent to e.m.a.vander.ven@vu.nl.

## Supplementary material

The supplementary material for this article can be found at http://doi.org/10.1017/gmh.2024.18.
