# Supplementary material for: Inequality on the frontline: A multi-country study on gender differences in mental health among healthcare workers during the COVID-19 pandemic
Source: Glob Ment Health (Camb). 2024 Mar 4;11:e34. doi: 10.1017/gmh.2024.18 (PMC10988139; doi:10.1017/gmh.2024.18)
Supplement: Czepiel et al. supplementary material [file S2054425124000189sup001.docx]

Supplementary material for the original paper entitled “Inequality on the frontline: A multi-country study on gender differences in mental health among healthcare workers during the COVID-19 pandemic” by Diana Czepiel^1,2†^, Clare McCormack^3†^, Andréa Tenório Correia da Silva^4,5^, Dominika Seblova^6^, Maria Francesca Moro^7^, Alexandra Restrepo-Henao^7,8^, Adriana Maldonado Martínez^7^, Oyeyemi Afolabi^9^, Lubna Alnasser^10^, Ruben Alvarado^11,12^, Hiroki Asaoka^13^, Olatunde Ayinde^14^, Arin Balalian^15^, Dinarte Ballester^16^, Josleen A. l. Barathie^17^, Armando Basagoitia^18^, Djordje Basic^2^, María Soledad Burrone^19^, Mauro Giovanni Carta^20^, Sol Durand-Arias^21^, Mehmet Eskin^22^, Eduardo Fernández-Jiménez^23,24,25^, Marcela Freytes^26^, Oye Gureje^14^, Anna Isahakyan^27^, Rodrigo Jaldo^26^, Elie G. Karam^17,28,29^, Dorra Khattech^30^, Jutta Lindert^31^, Gonzalo Martinez-Ales^32^, Franco Mascayano^7,33^, Roberto Mediavilla^34,35,36^, Javier Narvaez^7,37,38^, Aimee Nasser-Karam^17,28,29^, Daisuke Nishi^39^, Olusegun Olaopa^40^, Uta Ouali^30,41^, Victor Puac-Polanco^42, 43^, Dorian E. Ramírez^43^, Jorge Ramírez^12^, Eliut Rivera-Segarra^44^, Bart P. F. Rutten^45^, Julian Santaella-Tenorio^46^, Jaime C. Sapag^47,48,49^, Jana Šeblová^50,51^, María Teresa Solís Soto^52^, Maria Tavares-Cavalcanti^53^, Linda Valeri^54,32^, Marit Sijbrandij^2,55^, Ezra S. Susser^7,33^, Hans W. Hoek^56,1,7^, Els van der Ven^2*^ on behalf of the COVID‑19 HEalth caRe wOrkErS (HEROES) study group

^1^Parnassia Psychiatric Institute, Parnassia Groep, The Hague, the Netherlands

^2^Clinical, Neuro- and Developmental Psychology, Amsterdam Public Health Institute, Vrije Universiteit Amsterdam, the Netherlands

^3^Department of Child and Adolescent Psychiatry, New York University Langone Medical Center, New York, NY, USA

^4^University of São Paulo, São Paulo, Brazil

^5^ Faculty of Medicine Santa Marcelina, São Paulo, Brazil

^6^Second Faculty of Medicine, Charles University Prague, Prague, Czech Republic

^7^Department of Epidemiology, Columbia University Mailman School of Public Health, New York, NY, United States

^8^Epidemiology group, National school of public health, University of Antioquia, Colombia

^9^Department of Psychiatry, University College Hospital, Ibadan, Nigeria

^10^Population Health Research Section, King Abdullah International Medical Research Center (KAIMRC), King Saud bin Abdulaziz University for Health Sciences (KSAU-HS), Riyadh, Saudi Arabia

^11^Department of Public Health, School of Medicine, University of Valparaíso, Valparaiso, Chile

^12^School of Public Health, University of Chile, Santiago, Chile

^13^Department of Psychiatric Nursing, Graduate School of Medicine, The University of Tokyo, Tokyo, Japan

^14^Department of Psychiatry, University of Ibadan, Ibadan, Nigeria

^15^Question Driven Design and Analysis Group, New York, NY, USA

^16^University Hospital, Federal University of Rio Grande, Rio Grande, Brazil

^17^Institute for Development, Research, Advocacy and Applied Care (IDRAAC), Beirut, Lebanon

^18^Unidad de investigación, Consultora Salud Global Bolivia, Sucre, Bolivia

^19^Institute of Health Sciences, Universidad de O´Higgins, Rancagua, Chile

^20^Department of Medical Sciences and Public Health, University of Cagliari, Cagliari, Italy

^21^National Institute of Psychiatry Ramon de la Fuente Muñiz, Mexico City, Mexico

^22^Department of Psychology, Koç University, Istanbul, Turkey

^23^Department of Psychiatry, Clinical Psychology and Mental Health, La Paz University Hospital, Madrid, Spain

^24^Hospital La Paz Institute for Health Research (IdiPAZ), Madrid, Spain

^25^European University of Madrid, Madrid, Spain

^26^Social and Community Academic Unit, University of Chubut, Chubut, Argentina

^27^National Institute of Health Named After Academician S. Avdalbekyan, Yerevan, Armenia

^28^Department of Psychiatry and Clinical Psychology, University of Balamand Faculty of Medicine, Beirut, Lebanon

^29^Department of Psychiatry and Clinical Psychology, St George Hospital University Medical Center, Beirut, Lebanon

^30^Department Psychiatry A, Razi Hospital La Manouba, Manouba, Tunisia

^31^Faculty of Health and Social Work, University of Applied Sciences Emden / Leer, Emden, Germany

^32^Department of Epidemiology, Harvard T.H. Chan School of Public Health, Boston, MA, USA

^33^New York State Psychiatric Institute, New York, NY, United States

^34^Department of Psychiatry, Universidad Autónoma de Madrid, Madrid, Spain

^35^Centro de Investigación Biomédica en Red de Salud Mental (CIBERSAM), Carlos III Health Institute, Madrid, Spain

^36^Instituto de Investigación Sanitaria del Hospital Universitario La Princesa, Madrid, Spain

^37^Maestría en Epidemiología, División de Postgrados, El Bosque University, Bogota, Colombia

^38^Universidad Nacional de Colombia, Sede Bogotá, Facultad de Medicina, Departamento de Salud Pública, Bogotá, Colombia

^39^Department of Mental Health, Graduate School of Medicine, The University of Tokyo, Tokyo, Japan

^40^Department of Oral and Maxillofacial Surgery, University College Hospital, Ibadan, Nigeria

^41^Faculty of Medicine of Tunis, University of Tunis El Manar, Tunis, Tunisia

^42^Departments of Health Policy & Management and Epidemiology & Biostatistics, Downstate Health Sciences University, Brooklyn, NY, USA

^43^Faculty of Medical Sciences, University of San Carlos of Guatemala, Guatemala City, Guatemala

^44^School of Behavioral and Brain Sciences and Ponce Research Institute, Ponce Health Sciences University, Ponce, Puerto Rico

^45^Department of Psychiatry and Neuropsychology, School for Mental Health and Neuroscience, Maastricht University Medical Centre, Maastricht, the Netherlands

^46^Department of Clinical Epidemiology and Biostatistics, Pontifical Xavierian University, Bogota, Colombia

^47^Departments of Public Health and Family Medicine, Pontificia Universidad Católica de Chile, Santiago, Chile

^48^Dalla Lana School of Public Health, University of Toronto, Toronto, Canada

^49^Centre for Addiction and Mental Health, Ontario, Canada

^50^Emergency Department, Motol University Hospital, Prague, Czech Republic

^51^Czech Society for Emergency and Disaster Medicine of the Czech Medical Association J. E. Purkyně, Czech Republic

^52^Dirección de Investigación Ciencia y Tecnología, Universidad San Francisco Xavier de Chuquisaca, Bolivia

^53^School of Medicine and Psychiatric Institute, Federal University of Rio de Janeiro, Rio de Janeiro, Brazil

^54^Department of Biostatistics, Mailman School of Public Health, Columbia University, New York, NY, USA

^55^WHO Collaborating Center for Research and Dissemination of Psychological Interventions

^56^University Medical Center Groningen, University Center of Psychiatry, University of Groningen, Groningen, the Netherlands

*Corresponding author:

Els van der Ven

e.m.a.vander.ven@vu.nl

† Diana Czepiel and Clare McCormack contributed equally to this work and share first authorship.

Multilevel models consisted of two levels, with 32,410 individuals (individual-level) nested within 22 countries (country-level) and were fitted using maximum likelihood. The average country sample size was 1,473 (interquartile range: 167 – 5,502). Prior to performing the multilevel analyses, individual-level continuous predictors were group-mean centered, whereas the country-level predictors were grand-mean centered. A separate multilevel model was estimated for each mental health outcome, with the country as a random effect. Predictors of mental health at the individual level (contact with COVID-19 patients, considering PPE to be sufficient, experience of discrimination, interpersonal conflict or violence, support from colleagues and gender) were added as fixed effects, along with sociodemographic and clinical correlates. Interaction terms with gender were included for all individual-level predictors. At the country-level, the GII and COVID-19 mortality rates were added as fixed effects, along with cross-level interactions with gender. At the individual level, consistent with regression models, women were more likely to report depressive symptoms (OR = 1.61, 95% CI = 1.35 - 1.93) and psychological distress (OR = 1.44, 95% CI = 1.27 - 1.65) than men. Also, associations between all predictors and both mental health outcomes remained consistent with the previous regression models. At the country-level, no significant main effects were observed, but there was a statistically significant interaction between gender inequality and gender, suggesting that women living in countries with higher gender inequality reported fewer depressive symptoms (OR = .36, 95% CI = .21 - .62) and psychological distress (OR = .45, 95% CI = .29 - .70) than men.

In the random-effects model calculated to visualize gender differences in mental health outcomes across countries, the pooled effect size for depressive symptoms among women, as opposed to men, was 2.81 (95% CI = 2.77 - 2.86). For psychological distress it was 3.05 (95% CI = 2.85 - 3.27), suggesting that women reported significantly higher scores on both mental health outcomes compared to men across all countries. There seemed to be a mixed pattern regarding the association between psychological distress among women and a country’s gender inequality, with countries both with low and high gender inequality having effect sizes below or above the pooled effect size. Heterogeneity across countries, which was assessed by the I^2^ index, with higher scores being indicative of higher heterogeneity, was high for both psychological distress (I^2^ = 73.2%, p < .001) and depressive symptoms (I^2^ = 68.2%, p < .001).

The results of the regression models that were performed separately for physicians and nurses as sensitivity analyses can be found in Supplementary Table S6. Among nurses, men and women were equally likely to report depressive symptoms and psychological distress. Among women (n = 6,024), mental health outcomes were no longer significantly associated with insufficient PPE and COVID-19 mortality rates. Among men (n = 1,108), this was also the case for contact with COVID-19 patients and insufficient PPE and both mental health outcomes. In addition, the relation between support from colleagues and psychological distress was no longer statistically significant among men. In the subgroup of physicians, we observed a significant association between mortality rates and depressive symptoms (aOR = .58, 95% CI = .39 - .88) among women (n = 6,039), while this was no longer the case for the association with insufficient PPE. Among men (n = 3,597) the observed associations were in the same direction and of the same strength as in the main analyses.

**Supplementary Figure S1**


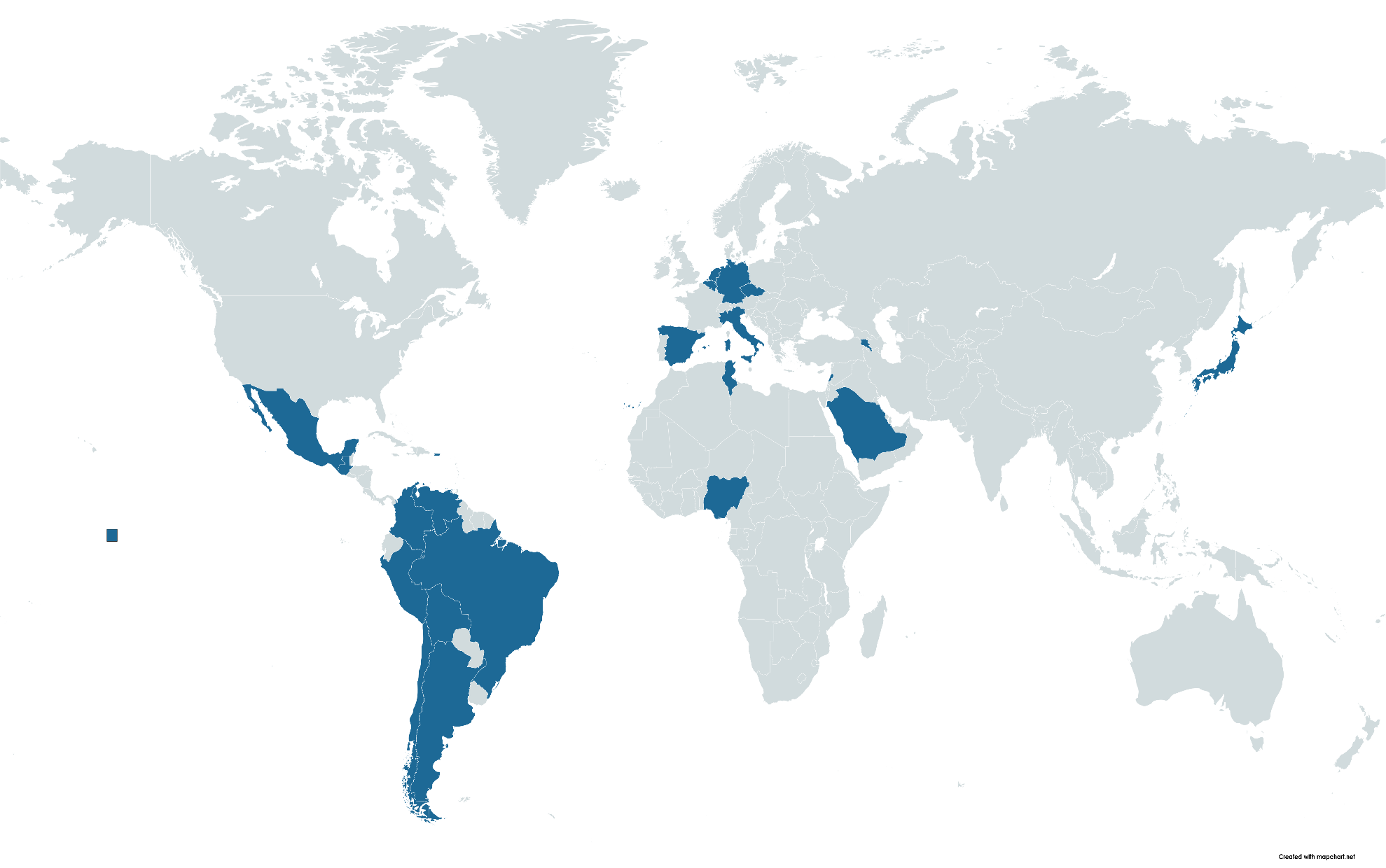


*Map of countries participating in the study*

| Supplementary Table S1  *Ad-hoc items created for the study and their response categories* | | | | | |
| --- | --- | --- | --- | --- | --- |
| Variable | Item | | | Response category | |
| Parents’ completed education | *“*What is the highest level of education completed by your mother (or principal caregiver 1)?”  *“*What is the highest level of education completed by your father (or principal caregiver 2)?” | | | Incomplete primary schooling/ primary school/ secondary school/ technical-professional training/ undergraduate degree (university training)/ postgraduate studies (master's, doctorate, medical specialty, etc.)/ does not apply | |
| Previous mental health problems | *“*Before the pandemic, did you have a mental health diagnosis?” | | | Yes/no/ I prefer not to respond | |
| Chronic physical illness | “Before the pandemic, did you have a chronic physical illness?” | | | Yes/no/ I prefer not to respond | |
| Being in contact with COVID-19 patients | *“*During the past week, have you been close to patients who were suspected or confirmed cases of COVID-19?” | | | Yes/no/ I don’t know | |
| Considering PPE to be sufficient | “Do you believe that the personal protective equipment you have access to is sufficient to avoid getting the virus?” | | | 0 = No, it is completely insufficient to 3 = Yes, it is sufficient | |
| Experience of discrimination ^a^ | “I have felt stigmatized or discriminated against as a health worker due to the COVID-19 pandemic” | | | 0 = Strongly disagree to 3 = Strongly agree | |
| Experience of interpersonal conflict ^a^ | “Since the beginning of the pandemic, have you experienced any problems with family members of patients with COVID-19?” | | | 0 = No, no problems to 4 = Yes, many problems | |
| Experience of violence ^a^ | “I have experienced violence due to being a health worker during the pandemic” | | | 0 = Strongly disagree to 3 = Strongly agree | |
| ^a^ These three variables were first dichotomized (experienced: “Strongly agree”, “Agree” / “Yes, one problem”, “Yes, some problems”, “Yes, many problems” vs. not experienced: “Strongly disagree”, “Disagree” / “No, no problems”). Then, the dichotomized variables were added creating a variable that indicated the number of types of interpersonal adversity HCWs have experienced. | | | | | |
| Supplementary Table S2  *Demographic and outcome variables by complete cases and non-complete cases* | | | | | |
|  | | Complete  cases  (n = 17,170) | Non-complete cases  (n = 15,240) | | χ^2^ / *Ws ^c^* |
| Gender, n (%) | |  |  | | 19.40, *p* < .001 |
| Women | | 12,593 (73.4) ^a^ | 10,574 (74.5) ^b^ | |  |
| Men | | 4,555 (26.5) ^a^ | 3,585 (25.2) ^b^ | |  |
| Other | | 18 (.1) ^a^ | 40 (.3) ^b^ | |  |
| Completed education, n (%) | |  |  | | 33.19, *p* < .001 |
| (Incomplete) primary school | | 126 (.7) ^a^ | 139 (1.0) ^b^ | |  |
| Secondary school | | 984 (5.7) ^a^ | 974 (6.9) ^b^ | |  |
| Technical-professional training | | 2,548 (14.9) ^a^ | 1,913 (13.6) ^b^ | |  |
| Undergraduate degree | | 6,091 (35.5) ^a^ | 5,092 (36.1) ^a^ | |  |
| Postgraduate studies | | 7,402 (43.2) ^a^ | 5,995 (42.5) ^a^ | |  |
| Occupation, n (%) | |  |  | | 47.22, *p* < .001 |
| Physicians | | 5,177 (30.3) ^a^ | 3,672 (28.7) ^b^ | |  |
| Nurses | | 4,868 (28.5) ^a^ | 3,514 (27.5) ^a^ | |  |
| Health technicians | | 3,550 (20.8) ^a^ | 2,581 (20.2) ^a^ | |  |
| Ancillary HCWs | | 1,864 (10.9) ^a^ | 1,545 (12.1) ^b^ | |  |
| Other HCWs | | 1,615 (9.5) ^a^ | 1,464 (11.5) ^b^ | |  |
| Chronic physical illness, n (%) | |  |  | | 12.09, *p* = .253 |
| No | | 10,921 (80.1) ^a^ | 5,535 (78.0) ^b^ | |  |
| Yes | | 2,718 (19.9) ^a^ | 1,560 (22.0) ^b^ | |  |
| Previous mental health problems, n (%) | |  |  | | 19.22, *p* = .001 |
| No | | 12,392 (92.4) ^a^ | 6,272 (90.6) ^b^ | |  |
| Yes | | 1,024 (7.6) ^a^ | 652 (9.4) ^b^ | |  |
| Age, Mdn (IQR) | | 39 (31-48) | 36 (30-46) | | 77304148.00, p < .001 |
| Psychological distress, Mdn (IQR) | | 13 (9-18) | 13 (9-18) | | 57851376.50, *p* = .228 |
| Depressive symptoms, Mdn (IQR) | | 5 (2-9) | 6 (2-10) | | 43112787.00, *p* < .001 |
| *Note.* Values not sharing the same subscript (^a, b^) are significantly different  Ancillary HCWs: e.g., non-clinical manager, administrator/secretary/admission, patient transportation, food/hospitality, cleaning staff, maintenance staff, security staff, student, statistician, analyst, IT, health information management  Other HCWs: e.g., clinical manager, psychologist, social worker, physical therapist, respiratory therapist, speech therapist, occupational therapist, first responder, midwife, dentist, dentist assistant, dietician, doctor assistant, epidemiologist/public health, pharmacist, community worker, primary attention worker, health promotion/prevention, health educator  ^c^ Value of the Chi square or Wilcoxon rank-sum test respectively | | | | | |

| Supplementary Table S3  *Sample size, age, gender, and occupation distribution per country and for the entire sample* | | | | | | | | | | |
| --- | --- | --- | --- | --- | --- | --- | --- | --- | --- | --- |
| Country | Sample  size | Age | Gender | | | Occupation | | | | |
|  |  |  | Women | Men | Other gender | Physicians | Nurses | Health technicians | Ancillary workers ^a^ | Other  HCWs ^b^ |
|  | n (%) | Mdn (IQR) | n (%) | n (%) | n (%) | n (%) | n (%) | n (%) | n (%) | n (%) |
| Argentina | 1,037 (3.2) | 40 (34-48) | 815 (79.8) | 203 (19.9) | 3 (0.3) | 232 (23.7) | 138 (14.1) | 35 (3.6) | 177 (18) | 398 (40.6) |
| Armenia | 570 (1.8) | 42 (31-50) | 465 (85.3) | 80 (14.7) | 0 (0) | 249 (49.8) | 136 (27.2) | 26 (5.2) | 32 (6.4) | 57 (11.4) |
| Belgium | 327 (1) | 40 (31-51) | 269 (82.5) | 56 (17.2) | 1 (0.3) | 46 (14.2) | 205 (63.3) | 9 (2.8) | 6 (1.8) | 58  (17.9) |
| Bolivia | 167 (0.5) | 35 (28-44) | 111 (68.1) | 50 (30.7) | 2 (1.2) | 55 (36.9) | 32 (21.5) | 18 (12.1) | 17 (11.4) | 27 (18.1) |
| Brazil | 3,246 (10) | 36 (30-43) | 2,613 (82.4) | 553 (17.5) | 4 (0.1) | 288 (9.5) | 459 (15.1) | 528 (17.3) | 591 (19.4) | 1,176 (38.7) |
| Chile | 2,495 (7.7) | 34 (30-43) | 1,797 (73.2) | 655 (26.7) | 3 (0.1) | 963 (40.1) | 246 (10.3) | 242 (10.1) | 228 (9.5) | 721 (30) |
| Colombia | 901 (2.8) | 39 (31-49) | 659 (73.6) | 237 (26.4) | 0 (0) | 185 (20.6) | 98 (10.9) | 22 (2.5) | 160 (17.8) | 432 (48.2) |
| Czech Republic | 1,801 (5.6) | 44 (35-53) | 1,349 (76.3) | 416 (23.5) | 44 (.02) | 472 (27.4) | 407 (23.6) | 125 (7.3) | 172 (10) | 546 (31.7) |
| Germany | 204 (0.6) | 39 (31-53) | 129 (63.6) | 74 (36.4) | 0 (0) | 29 (15) | 69 (35.7) | 3 (1.6) | 27 (14) | 65 (33.7) |
| Guatemala | 1,813 (5.6) | 33 (28-43) | 1,172 (66) | 601 (33.8) | 4 (0.2) | 644 (37.5) | 251 (14.6) | 159 (9.3) | 267 (15.5) | 397 (23.1) |
| Italy | 5,502 (17) | 44 (33-54) | 3,793 (70.9) | 1,550 (29) | 5 (0.1) | 1833 (35.6) | 649 (12.6) | 478 (9.3) | 217 (4.2) | 1,974 (38.3) |
| Japan | 810 (2.5) | 38 (31-49) | 215 (31.7) | 456 (67.3) | 7 (1) | 177 (26.4) | 242 (36.2) | 37 (5.5) | 74 (11.1) | 139 (20.8) |
| Lebanon | 768 (2.4) | 33 (27-47) | 530 (72.6) | 198 (27.1) | 2 (0.3) | 85 (13.7) | 207 (33.2) | 103 (16.5) | 127 (20.4) | 101 (16.2) |
| Mexico | 3,253 (10) | 37 (30-44) | 2,222 (70.2) | 939 (29.7) | 1 (0.1) | 1123 (37) | 752 (24.8) | 98 (3.2) | 314 (10.4) | 744 (24.6) |
| Netherlands | 683 (2.1) | 40 (31-51) | 533 (79.5) | 135 (20.2) | 2 (0.3) | 186 (29.4) | 192 (30.4) | 19 (3) | 60 (9.5) | 175 (27.7) |
| Nigeria | 459 (1.4) | 38 (32-45) | 262 (60) | 174 (39.8) | 1 (0.2) | 298 (64.2) | 75 (16.2) | 25 (5.4) | 36 (7.8) | 30 (6.5) |
| Peru | 3,670 (11.3) | 33 (28-40) | 2,742 (78.2) | 761 (21.7) | 5 (0.1) | 717 (21.7) | 931 (28.2) | 664 (20.1) | 209 (6.4) | 777 (23.6) |
| Puerto Rico | 266 (0.8) | 39 (31-48) | 193 (73.4) | 70 (26.6) | 0 (0) | 35 (14.1) | 54 (21.7) | 10 (4) | 50 (20.1) | 100 (40.1) |
| Saudi Arabia | 247 (0.8) | 35 (30-43) | 117 (48.4) | 122 (50.4) | 3 (1.2) | 77 (36.1) | 36 (16.9) | 17 (8) | 14 (6.6) | 69 (32.4) |
| Spain | 2,520 (7.8) | 41 (31-52) | 1,873 (78.2) | 515 (21.5) | 8 (0.3) | 764 (33.5) | 698 (30.7) | 208 (9.1) | 171 (7.5) | 438 (19.2) |
| Tunisia | 633 (1.9) | 34 (29-43) | 475 (76.7) | 142 (23) | 2 (0.3) | 373 (62.3) | 85 (14.2) | 45 (7.5) | 38 (6.3) | 58 (9.7) |
| Venezuela | 1,038 (3.2) | 41 (34-49) | 833 (84.4) | 153 (15.5) | 1 (0.1) | 153 (16.7) | 581 (63.6) | 19 (2.1) | 75 (8.2) | 86 (9.4) |
| Entire sample | 32,410 (100) | 38 (30-48) | 23,167 (73.9) | 8,140 (25.9) | 58 (0.2) | 8,849 (29.5) | 6,539 (21.8) | 2,903 (9.7) | 3,040 (10.1) | 8,656 (28.9) |
| *Note.* All percentages are valid percentages. The total number per gender and occupation differs due to missing data differing throughout survey sections ^a^ Non-clinical manager, administrator/secretary/admission, patient transportation, food/hospitality, cleaning staff, maintenance staff, security staff, student, statistician, analyst, IT, health information management ^b^ Clinical manager, psychologist, social worker, physical therapist, respiratory therapist, speech therapist, occupational therapist, first responder, midwife, dentist, dentist assistant, dietician, doctor assistant, epidemiologist/public health, pharmacist, community worker, primary attention worker, health promotion/prevention, health educator | | | | | | | | | | |

| Supplementary Table S4  *GII, average COVID-19 mortality rate and classification by income per country* | | | |
| --- | --- | --- | --- |
| Country | GII | COVID-19 mortality rate ^a^ | Country income ^b^ |
| Argentina | .287 | .52 | Upper-middle |
| Armenia | .216 | .33 | Upper-middle |
| Belgium | .048 | .03 | High |
| Bolivia | .418 | .43 | Lower-middle |
| Brazil | .39 | .28 | Upper-middle |
| Chile | .187 | .50 | High |
| Colombia | .424 | .74 | Upper-middle |
| Czech Republic | .12 | .47 | High |
| Germany | .073 | .11 | High |
| Guatemala | .481 | .13 | Upper-middle |
| Italy | .056 | .12 | High |
| Japan | .083 | .002 | High |
| Lebanon | .432 | .25 | Upper-middle |
| Mexico | .309 | .40 | Upper-middle |
| Netherlands | .025 | .04 | High |
| Nigeria | .68 | .003 | Lower-middle |
| Peru | .38 | .53 | Upper-middle |
| Puerto Rico | - | .18 | High |
| Saudi Arabia | .247 | .11 | High |
| Spain | .057 | .19 | High |
| Tunisia | .259 | .41 | Lower-middle |
| Venezuela | .492 | .02 | Upper-middle |
| *Note.* GII: Gender Inequality Index  ^a^ Average mortality rates during the recruitment period in each country calculated based on data by the Johns Hopkins Coronavirus Resource Center  ^b^ Based on the World Bank's fiscal year 2020 classification, with the exception of Venezuela, for which the year 2019 was utilized due to data unavailability. | | | |

| Supplementary Table S5  *Odds ratios and 95% confidence intervals [OR (95% CI)] for psychological distress for the entire sample and stratified by gender* | | | | | |
| --- | --- | --- | --- | --- | --- |
| Entire sample | | | | | |
|  | Unadjusted OR (95% CI) | | Adjusted OR (95% CI) | | |
| Gender (reference category = men) | 1.20** (1.14-1.26) | | 1.15** (1.09-1.22) | | |
|  | Women | | Men | | |
|  | Unadjusted OR (95% CI) | Adjusted OR (95% CI) |  | Unadjusted OR (95% CI) | Adjusted OR  (95% CI) |
| Work-related factors | | | | | |
| Contact with  COVID-19 patients | 1.50**  (1.41-1.59) | 1.34**  (1.25-1.44) |  | 1.59**  (1.44-1.76) | 1.41**  (1.25-1.59) |
| Insufficient PPE | 1.13**  (1.10-1.16) | 1.10**  (1.07-1.14) |  | 1.16**  (1.11-1.21) | 1.14**  (1.08-1.21) |
| Interpersonal factors | | | | | |
| Interpersonal   adversity | 1.46**  (1.42-1.50) | 1.38**  (1.33-1.43) |  | 1.41**  (1.34-1.48) | 1.29**  (1.22-1.37) |
| Unsupportive  colleagues | 1.39**  (1.34-1.44) | 1.33**  (1.28-1.39) |  | 1.26**  (1.19-1.33) | 1.19**  (1.11-1.27) |
| Country-level factors | | | | | |
| Gender inequality | .73**  (.62-.86) | .18**  (.14-.22) |  | .83  (.63-1.08) | .20**  (.14-.29) |
| COVID-19   mortality rates | 2.20**  (1.92-2.54) | 1.94**  (1.63-2.31) |  | 1.44*  (1.14-1.81) | 1.04  (.78-1.06) |
| Note. Adjusted OR = Odds ratio adjusted for age, mother’s education, father’s education, occupation, chronic physical illness and previous mental health problems and for all other predictors in Supplementary Table S5  * *p* < .05, ** *p* < .001 | | | | | |

| Supplementary Table S6  *Odds ratios ^a^ and 95% confidence intervals [OR (95% CI)] for depressive symptoms and psychological distress including country income stratified by gender* | | | | | |
| --- | --- | --- | --- | --- | --- |
|  | Depressive symptoms | |  | Psychological distress | |
|  | Women | Men |  | Women | Men |
| Work-related factors | | | | | |
| Contact with  COVID-19 patients | 1.56**  (1.43-1.70) | 1.37**  (1.16-1.62) |  | 1.37**  (1.28-1.47) | 1.44**  (1.27-1.62) |
| Insufficient PPE | 1.10**  (1.06-1.14) | 1.11*  (1.03-1.19) |  | 1.11**  (1.07-1.15) | 1.16**  (1.10-1.23) |
| Interpersonal factors | | | | | |
| Interpersonal   adversity | 1.50**  (1.44-1.56) | 1.55**  (1.44-1.67) |  | 1.38**  (1.33-1.43) | 1.29**  (1.21-1.37) |
| Unsupportive  colleagues | 1.42**  (1.36-1.49) | 1.56**  (1.44-1.69) |  | 1.32**  (1.27-1.37) | 1.19**  (1.11-1.27) |
| Country-level factors | | | | | |
| Gender inequality | .50*  (.30-.82) | .53  (.24-1.18) |  | .58*  (.38-.88) | .46*  (.25-.84) |
| COVID-19   mortality rates | .95  (.76-1.19) | .86  (.57-1.29) |  | 1.96**  (1.63-2.37) | 1.09  (.80-1.47) |
| Country income | .80**  (.74-.87) | .90  (.79-1.04) |  | .81**  (.76-.86) | .85*  (.76-.94) |
| ^a^ Odds ratio adjusted for age, mother’s education, father’s education, occupation, chronic physical illness and previous mental health problems and for all other predictors in Supplementary Table S6.  Unadjusted OR (95% CI) for country income are as follows: depressive symptoms among women, .94** (.91-.97); depressive symptoms among men, 1.10* (1.03-1.17); psychological distress among women, .96* (.94-.99); psychological distress among men, .97 (.93-1.02).  * *p* < .05, ** *p* < .001 | | | | | |

| Supplementary Table S7  *Odds ratios and 95% confidence intervals [OR (95% CI)] for depressive symptoms and psychological distress among physicians and nurses stratified by gender* | | | | | | | | | |
| --- | --- | --- | --- | --- | --- | --- | --- | --- | --- |
|  | Depressive symptoms | | | | | | | | |
|  | Physicians | | | |  | Nurses | | | |
|  | Unadjusted OR (95% CI) | | Adjusted OR (95% CI) | |  | Unadjusted OR (95% CI) | | Adjusted OR (95% CI) | |
| Gender (reference  category = men) | 1.77**  (1.59-1.97) | | 1.59**  (1.41-1.80) | |  | 1.05 (.89-1.23) | | 1.10  (.92-1.32) | |
|  | Physicians | | | |  | Nurses | | | |
|  | Women  (n = 6,039) | | Men  (n = 3,597) | |  | Women  (n = 6,024) | | Men  (n = 1,108) | |
|  | Unadjusted OR (95% CI) | Adjusted OR (95% CI) | Unadjusted OR (95% CI) | Adjusted OR (95% CI) |  | Unadjusted OR (95% CI) | Adjusted OR (95% CI) | Unadjusted OR (95% CI) | Adjusted OR (95% CI) |
| Work-related factors |  |  |  |  |  |  |  |  |  |
| Contact with  COVID-19 patients | 1.64**  (1.43-1.89) | 1.44**  (1.22-1.69) | 1.90**  (1.53-2.37) | 1.41*  (1.09-1.82) |  | 1.69**  (1.45-1.97) | 1.64**  (1.38-1.94) | 1.69*  (1.17-2.45) | 1.21  (.79-1.84) |
| Insufficient PPE | 1.18**  (1.11-1.25) | 1.06  (.98-1.14) | 1.26**  (1.15-1.37) | 1.12*  (1.01-1.26) |  | 1.04  (.98-1.10) | 1.07  (.99-1.15) | 1.09  (.95-1.26) | 1.05  (.88-1.24) |
| Interpersonal factors |  |  |  |  |  |  |  |  |  |
| Interpersonal adversity | 1.54**  (1.44-1.63) | 1.44**  (1.34-1.55) | 1.86**  (1.70-2.03) | 1.56**  (1.41-1.74) |  | 1.50**  (1.41-1.60) | 1.46**  (1.36-1.58) | 1.74**  (1.50-2.02) | 1.66**  (1.39-2.00) |
| Unsupportive  colleagues | 1.49**  (1.38-1.60) | 1.41**  (1.34-1.55) | 1.54**  (1.39-1.71) | 1.43**  (1.26-1.61) |  | 1.37**  (1.27-1.48) | 1.36**  (1.24-1.48) | 1.60**  (1.34-1.91) | 1.50**  (1.21-1.86) |
| Country-level factors |  |  |  |  |  |  |  |  |  |
| Gender inequality | 1.66*  (1.13-2.42) | .38**  (.23-.64) | 2.40*  (1.43-4.02) | .50*  (.25-.99) |  | .40**  (.28-.58) | .09**  (.05-.15) | 1.31  (.55-3.14) | .16* (.05-55) |
| COVID-19 mortality rates | 1.13  (.80-1.57) | .58*  (.39-.88) | 1.71*  (1.08-2.71) | .81  (.46-1.42) |  | 1.45*  (1.06-2.00) | 1.01  (.68-1.51) | 2.63*  (1.20-5.77) | 1.19  (.42-3.35) |
|  | Psychological distress | | | | | | | | |
|  | Physicians | | | |  | Nurses | | | |
|  | Unadjusted OR (95% CI) | | Adjusted OR (95% CI) | |  | Unadjusted OR (95% CI) | | Adjusted OR (95% CI) | |
| Gender (reference  category = men) | 1.41**  (1.29-1.53) | | 1.28**  (1.16-1.41) | |  | 1.03 (.90-1.18) | | 1.08  (.91-1.26) | |
|  | Physicians | | | |  | Nurses | | | |
|  | Women  (n = 6,039) | | Men  (n = 3,597) | |  | Women  (n = 6,024) | | Men  (n = 1,108) | |
|  | Unadjusted OR (95% CI) | Adjusted OR (95% CI) | Unadjusted OR (95% CI) | Adjusted OR (95% CI) |  | Unadjusted OR (95% CI) | Adjusted OR (95% CI) | Unadjusted OR (95% CI) | Adjusted OR (95% CI) |
| Work-related factors |  |  |  |  |  |  |  |  |  |
| Contact with  COVID-19 patients | 1.56**  (1.39-1.75) | 1.37** (1.20-1.57) | 1.84**  (1.57-2.15) | 1.65** (1.38-1.98) |  | 1.44**  (1.27-1.63) | 1.45** (1.27-1.66) | 1.17  (.88-1.56) | 1.09  (.79-1.50) |
| Insufficient PPE | 1.23**  (1.17-1.30) | 1.19**  (1.11-1.27) | 1.18**  (1.10-1.27) | 1.18** (1.08-1.28) |  | 1.02  (.97-1.08) | 1.05  (.98-1.11) | 1.05  (.93-1.18) | 1.05  (.91-1.21) |
| Interpersonal factors |  |  |  |  |  |  |  |  |  |
| Interpersonal adversity | 1.49**  (1.41-1.58) | 1.36**  (1.27-1.45) | 1.46**  (1.35-1.56) | 1.29**  (1.18-1.40) |  | 1.34**  (1.27-1.42) | 1.34** (1.25-1.43) | 1.31**  (1.15-1.48) | 1.33**  (1.14-1.55) |
| Unsupportive  colleagues | 1.43**  (1.34-1.53) | 1.37** (1.26-1.47) | 1.29**  (1.19-1.40) | 1.21** (1.10-1.33) |  | 1.24**  (1.16-1.32) | 1.26** (1.16-1.36) | 1.19*  (1.02-1.38) | 1.18  (.99-1.42) |
| Country-level factors |  |  |  |  |  |  |  |  |  |
| Gender inequality | 1.49*  (1.07-2.09) | .29** (.19-.46) | 1.29  (.85-1.89) | .25** (.15-.43) |  | .34**  (.25-.47) | .13**  (.08-.19) | .46*  (.22-.95) | .14**  (.05-.37) |
| COVID-19 mortality rates | 1.89**  (1.42-2.53) | 1.40  (.98-1.98) | 1.64*  (1.56-2.32) | 1.12  (.74-1.70) |  | 1.15  (.88-1.51) | .96  (.70-1.33) | .91  (.47-1.77) | .71  (.31-1.64) |
| Note. Adjusted OR = Odds ratio adjusted for age, mother’s education, father’s education, occupation, chronic physical illness and previous mental health problems and for all other predictors in Supplementary Table S7 * *p* < .05, ** *p* < .001 | | | | | | | | | |

| Supplementary Table S8  *Multilevel models for depressive symptoms and psychological distress* | | | | | |
| --- | --- | --- | --- | --- | --- |
|  | Depressive symptoms | |  | Psychological distress | |
| Variables | OR | CI |  | OR | CI |
| Intercept | .07** | .05 - .09 |  | .33** | .22 - .50 |
| Individual level |  |  |  |  |  |
| Age | .99** | .98 -.99 |  | .99** | .98 - .99 |
| Mother’s education (ref. cat. = postgraduate studies) |  |  |  |  |  |
| Incomplete primary   school | .88 | .74 - 1.05 |  | .91 | .78 - 1.06 |
| Primary school | .92 | .79 - 1.07 |  | .93 | .82 - 1.06 |
| Secondary school | .89 | .77 - 1.02 |  | .88* | .78 - .98 |
| Technical- professional   training | .93 | .81 - 1.07 |  | .93 | .82 - 1.05 |
| Undergraduate studies | 1.09 | .95 - 1.25 |  | .96 | .85 - 1.08 |
| N/A | .77 | .58 - 1.03 |  | .73 | .58 - .94 |
| Father’s education (ref. cat. = postgraduate studies) |  |  |  |  |  |
| Incomplete primary   school | .98 | .82 - 1.16 |  | .94 | .81 - 1.09 |
| Primary school | .98 | .85 - 1.13 |  | .91 | .81 - 1.03 |
| Secondary school | .99 | .87 - 1.13 |  | .90 | .81 - 1.01 |
| Technical- professional   training | .98 | .86 - 1.11 |  | .94 | .84 - 1.05 |
| Undergraduate studies | .91 | .80 - 1.03 |  | 1.02 | .92 - 1.14 |
| N/A | 1.13 | .91 - 1.41 |  | .92 | .76 - 1.12 |
| Current occupation (ref. cat. = other HCWs ^a^) |  |  |  |  |  |
| Physicians | 1.01 | .92 - 1.11 |  | 1.28** | 1.18 - 1.38 |
| Nurses | 1.09 | .99 - 1.20 |  | .99 | .92 - 1.08 |
| Health technicians | .99 | .87 - 1.12 |  | .85* | .76 -.95 |
| Ancillary HCWs ^b^ | 1.11 | .98 - 1.26 |  | 1.00 | .90 - 1.12 |
| Chronic physical illness  (ref. cat. = none) | 1.37** | 1.27 - 1.48 |  | 1.33** | 1.24 - 1.42 |
| Previous mental health problems (ref. cat. = none) | 2.77** | 2.50 - 3.06 |  | 2.35** | 2.12 - 2.60 |
| Contact with COVID-19 patients (ref. cat. = no contact) | 1.36* | 1.42 - 1.63 |  | 1.19* | 1.04 - 1.36 |
| Insufficient PPE | 1.25** | 1.14 - 1.36 |  | 1.27** | 1.18 - 1.36 |
| Experienced interpersonal adversity (ref. cat. = none) |  |  |  |  |  |
| 1 | 1.86** | 1.56 - 2.22 |  | 1.65** | 1.45 - 1.89 |
| 2 | 2.81** | 2.26 - 3.50 |  | 2.63** | 2.23 - 3.12 |
| 3 | 4.09** | 3.12 - 5.37 |  | 2.98** | 2.40 - 3.69 |
| Unsupportive colleagues | 1.53** | 1.40 - 1.68 |  | 1.52** | 1.41 - 1.65 |
| Gender; women (ref. cat. = men) | 1.61** | 1.35 - 1.93 |  | 1.44** | 1.27 - 1.65 |
| Contact with COVID-19 patients *  gender; women | 1.05 | .87 - 1.26 |  | 1.03 | .88 - 1.19 |
| Insufficient PPE * gender; women | .94 | .87 - 1.03 |  | .94 | .87 - 1.01 |
| 1 type of interpersonal adversity *  gender; women | .84 | .69 - 1.01 |  | .96 | .82 - 1.12 |
| 2 types of interpersonal adversity *  gender; women | .87 | .69 - 1.09 |  | .89 | .73 - 1.07 |
| 3 types of interpersonal adversity *  gender; women | .83 | .63 - 1.11 |  | 1.06 | .82 - 1.36 |
| Unsupportive colleagues * gender; women | .94 | .86 - 1.04 |  | .95 | .87 - 1.03 |
| Country level |  |  |  |  |  |
| Gender inequality | 1.21 | .41 - 3.57 |  | .49 | .06 - 4.04 |
| COVID-19 mortality rates | 2.19 | .88 - 5.44 |  | 3.32 | .54 - 20.34 |
| Gender inequality *  gender; women | .36** | .21 -.62 |  | .45** | .29 -.70 |
| COVID-19 mortality rates *  gender; women | 1.30 | .83 - 2.03 |  | 1.01 | .69 - 1.47 |
| Note. Ref. cat. = reference category, PPE = personal protective equipment, N/A = not applicable (HCWs who could not answer the question about their mother’s or father’s education level because it was not applicable to them)  ^a^ Other HCWs: e.g., clinical manager, psychologist, social worker, physical therapist, respiratory therapist, speech therapist, occupational therapist, first responder, midwife, dentist, dentist assistant, dietician, doctor assistant, epidemiologist/public health, pharmacist, community worker, primary attention worker, health promotion/prevention, health educator  ^b^ Ancillary HCWs: e.g., non-clinical manager, administrator/secretary/admission, patient transportation, food/hospitality, cleaning staff, maintenance staff, security staff, student, statistician, analyst, IT, health information management  * *p* < .05, ** *p* < .001 | | | | | |

**Supplementary Figure S2**


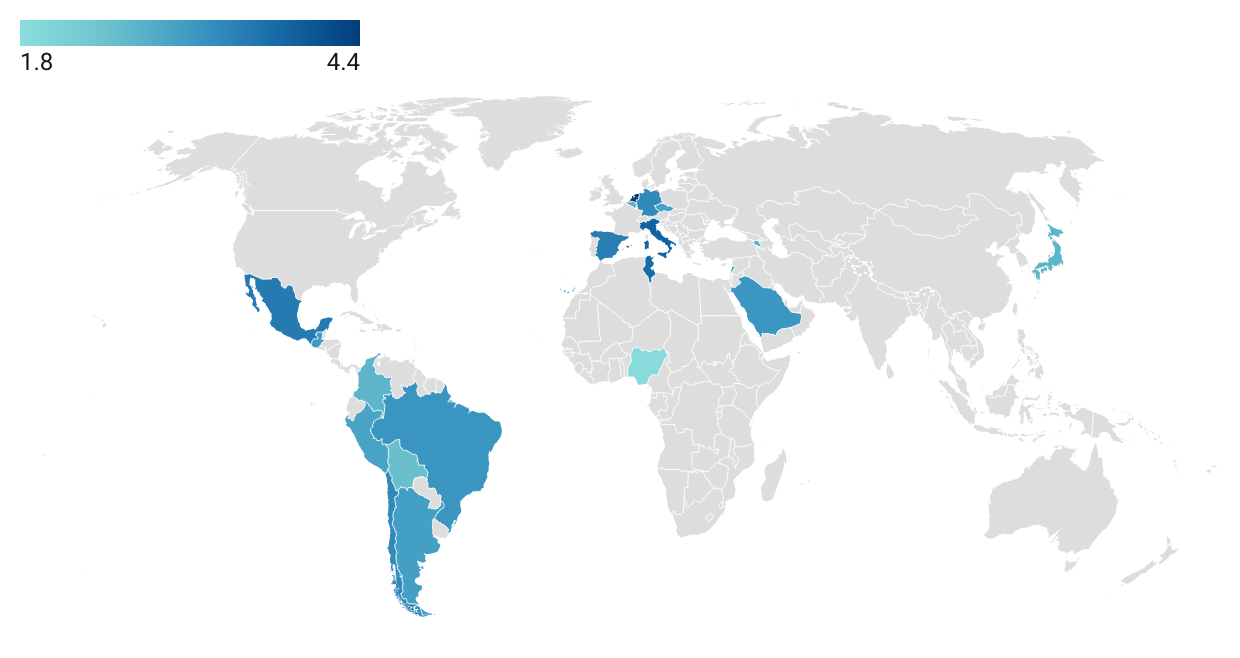

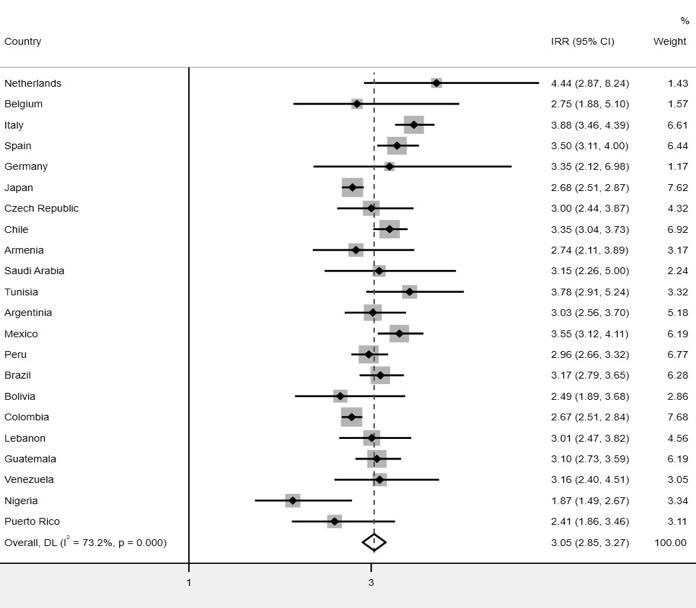


*Incidence rate ratio (IRR) of psychological distress for women compared to men across countries (A);* the intensity of the color corresponds to the IRR, with darker shades indicating higher IRR values. *(B) IRRs listed according to the Gender Inequality Index of countries in ascending order. IRR’s range from 1.8 to 4.4.*

| Supplementary Table S9  *The HEORES study group* | |
| --- | --- |
| Argentina |  |
|  | Marcela Freytes and Rodrigo Jaldo  Social and Community Academic Unit, Universidad de Chubut |
| Armenia |  |
|  | Arin Allahverdi Balalian  Department of Epidemiology, Mailman School of Public Health, Columbia University |
|  | Anna Isahakyan and Alexander Bazarchyan  National Institute of Health Named After Academician S. Avdalbekyan |
|  | Khachatur Gasparyan  Yerevan State Medical University |
| Australia ^a^ |  |
|  | Sonja Memedovic  National Drug and Alcohol Research Centre  University of New South Wales |
|  | Clare McCormack  Center for Science and Society Columbia University |
| Belgium |  |
|  | Lydia Gisle  Epidemiology and Public Health Sciensano |
| Bolivia |  |
|  | Armando Basagoitia  Unidad de investigación, Consultora Salud Global Bolivia  María Teresa Solís Soto  Universidad San Francisco Xavier de Chuquisaca |
| Brazil |  |
|  | Dinarte Ballesteres  Hospital Universitário, Universidade Federal do Rio Grande |
|  | Maria Tavares Calvanti  Faculty of Medicine Universidade Federal do Rio de Janeiro |
|  | Andrea Tenorio  Faculty of Medicine, University of São Paulo |
| Chile |  |
|  | Jorge Ramírez, Gonzalo Soto, Rubén Alvarado, Sara Schiling, Sebastián Alarcón, Jorge Caro, and Alex Benavides  School of Public Health, Universidad de Chile |
|  | Rodrigo Goycolea, Eric Tapia, and Margarita Corté  Universidad Central de Chile |
|  | María Soledad Burrone  Universidad de O'Higgins |
|  | Gonzalo Cuadra and Josefina Huneeus  Colegio Médico de Chile |
|  | Jaime Sapag, Carolina Traub, and Paula Bedregal  Pontificia Universidad Católica de Chile |
|  | Sebastián Villarroel González  Servicio de Salud del Reloncaví |
|  | Antonia Aguirre  Corporación Municipal de Renca |
|  | Ítalo Lanio  Ministerio de Salud |
|  | Thamara Tapia  University College London |
|  | Kasim Allel  London School of Hygiene and Tropical Medicine, |
| Colombia |  |
|  | Alexandra Restrepo-Henao  Universidad de Antioquia, Columbia University |
|  | Javier Narvaez  Department of Epidemiology, Mailman School of Public Health, Columbia University |
|  | Julián Santaella-Tenorio  Department of Clinical Epidemiology and Biostatistics, Pontificia Universidad Javeriana |
|  | Adriana Maldonado  Department of Epidemiology, Mailman School of Public Health, Columbia University |
| Czech Republic |  |
|  | Nika Seblova  Second Faculty of Medicine, Charles University Prague, Prague, Czech Republic |
|  | Jana Seblova  Second Faculty of Medicine, Charles University Prague, Prague, Czech Republic Emergency Department, Motol University Hospital, Prague, Czech Republic |
| Ecuador ^a^ |  |
|  | Sandra Muñóz Ministerio de Salud Pública, Guayaquil |
|  | Andrés Peralta Agència de Salut Pública de Barcelona, Barcelona, Spain |
| Germany |  |
|  | Jutta Lindert  University of Applied Sciences Emden/Leer, Emden |
| Guatemala |  |
|  | Dorian E. Ramírez, Aida Barrera-Pérez, and Erwin Calgua  Facultad de Ciencias Médicas, Universidad de San Carlos de Guatemala |
|  | María Alejandra Paniagua-Ávila  Department of Epidemiology, Mailman School of Public Health, Columbia University |
|  | Ana Peralta-Garcia  Department of Epidemiology and Biostatistics, Dornsife School of Public Health, Drexel University |
|  | Victor Puac-Polanco  Departments of Health Policy & Management and Epidemiology & Biostatistics, Downstate Health Sciences University and Facultad de Ciencias Médicas, Universidad de San Carlos de Guatemala |
| India ^a^ |  |
|  | Abhijit Nadkarni, Anant Bhan, Urvita Bhatia, and Madhavi Roy Addictions Research Group, Sangath, Goa |
|  | Aravind Pillai Columbia Alumni Association, Columbia University |
| Italy |  |
|  | Maria Francesca Moro  Department of Epidemiology, Mailman School of Public Health, Columbia University |
|  | Mauro Giovanni Carta  Università degli studi di Cagliari |
| Japan |  |
|  | Daisuke Nishi  Department of Mental Health, School of Public Health, The University of Tokyo |
|  | Hiroki Asaoka  Department of Psychiatric Nursing, Graduate School of Medicine, The University of Tokyo |
|  | Norito Kawakami  Department of Digital Mental Health, Graduate School of Medicine, The University of Tokyo |
| Lebanon |  |
|  | Elie Karam, Josleen Al Barathie, Georges Karam, and Aimee Karam  Institute for Development Research Advocacy and Applied Care, Beirut |
| Mexico |  |
|  | Sol Durand-Arias  Instituto Nacional de Psiquiatría Ramón de la Fuente Muñiz |
|  | Jaime Carmona-Huerta  Instituto Jalisciense de Salud Mental, Jalisco |
| Nigeria |  |
|  | Oye Gureje, Olatunde Ayinde, and Oyeyemi Afolabi  Department of Psychiatry, University College Hospital, Ibadan |
|  | Olusegun Olaopa  Department of Oral and Maxillofacial Surgery, University College Hospital, Ibadan |
| North Macedonia ^a^ |  |
|  | Andre Dwork  Department of Pathology and Cell Biology, Columbia University |
|  | Gorazd Rosoklija Department of Psychiatry, Columbia University |
| Peru |  |
|  | Andrew Dwork  Dirección de Salud Mental del Ministerio de Salud |
| Poland ^a^ |  |
|  | Jakub Bil Ecological Psychiatry Commission of Polish Psychiatric Association |
| Puerto Rico |  |
|  | Eliut Rivera  School of Behavioral and Brain Sciences, Ponce Health Sciences University |
| Saudi Arabia |  |
|  | Lubna Alnasser  Department of Epidemiology, Mailman School of Public Health, Columbia University and King Abdullah International Medical Research Center, King Saud Bin Abdulaziz University for Health Sciences |
|  | Suliman Alghnam  King Abdullah International Medical Research Center, King Saud Bin Abdulaziz University for Health Sciences |
| South Africa ^a^ |  |
|  | Landon Myer and Kirsty Brittain  School of Public Health & Family Medicine University of Cape Town |
| Spain |  |
|  | Marife Bravo-Ortiz, Eduardo Fernández Jiménez, and Roberto Mediavilla Torres  Hospital Universitario La Paz, Madrid |
|  | Gonzalo Martínez-Alés  Department of Epidemiology, Mailman School of Public Health, Columbia University |
| The Netherlands |  |
|  | Els van der Ven  Faculty of Behavioural and Movement Sciences, Vrije Universiteit, Amsterdam |
| Tunisia |  |
|  | Uta Ouali, Dorra Khattech, and Fethi Nacef  Psychiatry department A, Razi Hospital, Faculty of Medicine of Tunis, University of Tunis El Manar |
|  | Amira Jamoussi  Medical Intensive Care, Abderrahmane Memi Hospital, Faculty of Medicine of Tunis, University of Tunis El Manar |
| Turkey ^a^ |  |
|  | Mehmet Eskin Department of Psychology, Koc University |
| Uruguay ^a^ |  |
|  | Luis Giménez Facultad de Psicología Universidad de la República |
| Venezuela |  |
|  | Ana María Rodríguez  Instituto Altos Estudios Dr Arnoldo Gabaldon |
| *Note.* These countries encountered diverse challenges that ultimately hindered data collection, despite their involvement in the study's conceptualization. These challenges included issues such as not obtaining ethical approval (e.g., India), grappling with significant internal crises (e.g., South Africa), or facing difficulties in enrolling participants (e.g., Australia). | |
