# Supplementary material for: Inequality on the frontline: A multi-country study on gender differences in mental health among healthcare workers during the COVID-19 pandemic
Source: Glob Ment Health (Camb). 2024 Mar 4;11:e34. doi: 10.1017/gmh.2024.18 (PMC10988139; doi:10.1017/gmh.2024.18)
Supplement: Peer review history

# Editor report: Recommendation: Inequality on the frontline: A multi-country study on gender differences in mental health among healthcare workers during the COVID-19 pandemic — R0/PR2

**DOI:** 10.1017/gmh.2024.18.pr2

**Affiliations:** The George Institute for Global Health India, India

I liked the paper and the analyses done. What was missing was data by WB income category of countries. While GII is a good index may be a supplementary table highlighting differences across gender by country income groups is worth exploring and indicating.

Also some of the current countries listed in the map does not tally with the HEROES research group. Eg I do not see India, though we have teams from India listed in the HEROES group. Please clarify.

The group Middle East and Asia is just not a homogeneous group in any sense.

Pl address comments made specifically by one reviewer.

# Editor report: Decision: Inequality on the frontline: A multi-country study on gender differences in mental health among healthcare workers during the COVID-19 pandemic — R0/PR3

**DOI:** 10.1017/gmh.2024.18.pr3

**Affiliations:** London School of Hygiene & Tropical Medicine, United Kingdom of Great Britain and Northern Ireland

No accompanying comment.

# Editor report: Recommendation: Inequality on the frontline: A multi-country study on gender differences in mental health among healthcare workers during the COVID-19 pandemic — R1/PR5

**DOI:** 10.1017/gmh.2024.18.pr5

**Affiliations:** The George Institute for Global Health India, India

No accompanying comment.

# Editor report: Decision: Inequality on the frontline: A multi-country study on gender differences in mental health among healthcare workers during the COVID-19 pandemic — R1/PR6

**DOI:** 10.1017/gmh.2024.18.pr6

**Affiliations:** London School of Hygiene & Tropical Medicine, United Kingdom of Great Britain and Northern Ireland

No accompanying comment.
